# Supplementary material for: Swedish multimodal cohort of patients with anxiety or depression treated with internet-delivered psychotherapy (MULTI-PSYCH)
Source: BMJ Open. 2023 Oct 4;13(10):e069427. doi: 10.1136/bmjopen-2022-069427 (PMC10551950; doi:10.1136/bmjopen-2022-069427)
Supplement: Author's manuscript [file bmjopen-2022-069427.draft_revisions.pdf]

BMJ Open

BMJ Open is committed to open peer review. As part of this commitment we make the peer review history of every article we publish publicly available.

When an article is published we post the peer reviewers' comments and the authors' responses online. We also post the versions of the paper that were used during peer review. These are the versions that the peer review comments apply to.

The versions of the paper that follow are the versions that were submitted during the peer review process. They are not the versions of record or the final published versions. They should not be cited or distributed as the published version of this manuscript.

BMJ Open is an open access journal and the full, final, typeset and author-corrected version of record of the manuscript is available on our site with no access controls, subscription charges or pay-per-view fees (http://bmjopen.bmj.com).

If you have any questions on BMJ Open's open peer review process please email info.bmjopen@bmj.com

BMJ Open

Cohort profile: A Swedish multimodal database of patients with anxiety and depression treated with internet-delivered psychotherapy (MULTI-PSYCH)

Journal:	*BMJ Open*
Manuscript ID	bmjopen-2022-069427
Article Type:	Cohort profile
Date Submitted by the Author:	07-Dec-2022
Complete List of Authors:	Boberg, Julia; Karolinska Institute Department of Clinical Neuroscience<br>Kaldo, V; Karolinska Institute Department of Clinical Neuroscience; Linnaeus University Faculty of Health and Life Sciences, Department of Psychology<br>Mataix-Cols, David; Karolinska Institute Department of Clinical Neuroscience<br>Crowley, James; The University of North Carolina at Chapel Hill Department of Genetics; The University of North Carolina at Chapel Hill Department of Psychiatry<br>Roelstraete, Bjorn; Karolinska Institute Department of Medical Epidemiology and Biostatistics<br>Halvorsen, Matthew; The University of North Carolina at Chapel Hill Department of Genetics<br>Forsell, Erik; Karolinska Institute Department of Clinical Neuroscience<br>Isacsson, Nils; Karolinska Institute Department of Clinical Neuroscience<br>Sullivan, Patrick F.; Karolinska Institute Department of Medical Epidemiology and Biostatistics; The University of North Carolina at Chapel Hill Department of Genetics<br>Svanborg, Cecilia; Karolinska Institute Department of Clinical Neuroscience<br>Andersson, Evelyn; Karolinska Institute Department of Clinical Neuroscience<br>Lindefors, Nils; Karolinska Institute Department of Clinical Neuroscience<br>Kravchenko, Olly; Karolinska Institute Department of Clinical Neuroscience<br>Mattheisen, Manuel; University of Aarhus Department of Biomedicine<br>Danielsdottir, Hilda; Karolinska Institute Department of Clinical Neuroscience<br>Ivanova, Ekaterina; Karolinska Institute Department of Clinical Neuroscience<br>Boman, Magnus; Karolinska Institute Department of Learning Informatics Management and Ethics<br>Fernández de la Cruz, Lorena; Karolinska Institute Department of Clinical Neuroscience<br>Wallert, John; Karolinska Institute Department of Clinical Neuroscience<br>Rück, Christian; Karolinska Institute Department of Clinical Neuroscience
Keywords:	Anxiety disorders < PSYCHIATRY, Depression & mood disorders < PSYCHIATRY, GENETICS, EPIDEMIOLOGY

I, the Submitting Author has the right to grant and does grant on behalf of all authors of the Work (as defined in the below author licence), an exclusive licence and/or a non-exclusive licence for contributions from authors who are: i) UK Crown employees; ii) where BMJ has agreed a CC-BY licence shall apply, and/or iii) in accordance with the terms applicable for US Federal Government officers or employees acting as part of their official duties; on a worldwide, perpetual, irrevocable, royalty-free basis to BMJ Publishing Group Ltd ("BMJ") its licensees and where the relevant Journal is co-owned by BMJ to the co-owners of the Journal, to publish the Work in this journal and any other BMJ products and to exploit all rights, as set out in our licence.

The Submitting Author accepts and understands that any supply made under these terms is made by BMJ to the Submitting Author unless you are acting as an employee on behalf of your employer or a postgraduate student of an affiliated institution which is paying any applicable article publishing charge ("APC") for Open Access articles. Where the Submitting Author wishes to make the Work available on an Open Access basis (and intends to pay the relevant APC), the terms of reuse of such Open Access shall be governed by a Creative Commons licence – details of these licences and which Creative Commons licence will apply to this Work are set out in our licence referred to above.

Other than as permitted in any relevant BMJ Author's Self Archiving Policies, I confirm this Work has not been accepted for publication elsewhere, is not being considered for publication elsewhere and does not duplicate material already published. I confirm all authors consent to publication of this Work and authorise the granting of this licence.

For peer review only

Cohort profile: A Swedish multimodal database of patients with anxiety and depression treated with internet-delivered psychotherapy (MULTI-PSYCH)

Julia Boberg[1,] Viktor Kaldo[1,2], David Mataix-Cols[1], James J Crowley[1,4,5], Bjorn Roelstraete[3], Matthew W Halvorsen[4], Erik Forsell[1], Nils Hentati Isacsson[1], Patrick F Sullivan[3,4,5], Cecilia Svanborg[1], Evelyn Andersson Hagen[1], Nils Lindefors[1], Olly Kravchenko[1], Manuel Mattheisen[1,8,9], Hilda Björk Daníelsdóttir[1], Ekaterina Ivanova[1], Magnus Boman[6,7], Lorena Fernández de la Cruz[1], John Wallert[1*], Christian Rück[1*]
*shared last author

Corresponding author: Julia Boberg, julia.boberg@ki.se, M46, Psychiatry Southwest, Karolinska University Hospital, SE-141 86 Huddinge, Sweden.

[1]Centre for Psychiatry Research, Department of Clinical Neuroscience, Karolinska Institutet, & Stockholm Health Care Services, Region Stockholm, Stockholm, Sweden
[2]Department of Psychology, Faculty of Health and Life Sciences, Linnaeus University, Växjö, Sweden.
[3]Department of Medical Epidemiology and Biostatistics, Karolinska Institutet, Stockholm, Sweden.
[4]Department of Genetics, University of North Carolina, Chapel Hill, North Carolina.
[5]Department of Psychiatry, University of North Carolina, Chapel Hill, North Carolina.
[6]Department of Learning, Informatics, Management and Ethics, Karolinska Institutet, Stockholm, Sweden.
[7]Department of Computer and Software Systems, School of EECS, KTH, Stockholm, Sweden.
[8]Department of Biomedicine, Aarhus University, Aarhus, Denmark.
[9]Department of Psychiatry, Psychosomatics and Psychotherapy, University of Würzburg, Würzburg, Germany.

Word count: 3605

For peer review only

Abstract

Purpose: Depression and anxiety afflict millions worldwide causing considerable disability every year. MULTI-PSYCH is a longitudinal cohort of genotyped and richly phenotyped individuals with depression and anxiety disorders who have undergone highly structured treatment with internet-based cognitive behaviour therapy (ICBT). The overarching purpose of MULTI-PSYCH is to improve risk stratification, outcome prediction, and secondary prevention for individuals with depression and anxiety disorders. The MULTI-PSYCH cohort is a precision medicine initiative that combines genetic, clinical, and nationwide register data.

Participants: The cohort includes 2668 clinically well-characterised adults with major depressive disorder (MDD) (n=1300), social anxiety disorder (n=640) or panic disorder (n=728) assessed before, during, and after 12 weeks of internet-based cognitive behaviour (ICBT) at the Internet psychiatry clinic in Stockholm, Sweden. All participants provided blood samples that were genotyped for single nucleotide polymorphisms (SNPs). Clinical and genetic data were then linked to several Swedish registers containing a wide range of variables, spanning from birth to up to 10 years after the end of treatment. Some examples include perinatal complications, school grades, psychiatric and somatic comorbidity, dispensed medications, health care and social benefits, and income. Long-term follow-up data linkage from registers will continue until 2029.

Findings to date: Initial uses of MULTI-PSYCH include the discovery of an association between PRS for autism spectrum disorder and response to ICBT, the development of a machine learning model for baseline prediction of remission status after ICBT in MDD, and data contributions to genome wide association (GWA) studies for CBT outcomes. Other projects have been launched or are in the planning stage.

Future plans: The MULTI-PSYCH cohort provides a unique infrastructure to study predictors or short-term treatment outcomes, and longer-term medical and socioeconomic outcomes in patients with depression or anxiety disorders. MULTI-PSYCH is well-positioned for, and encourages research collaboration

**Strengths and limitations of this study**
• MULTI-PSYCH is a unique integration of clinical, genetic and register data<br>• Long-term follow-up allows for the study of outcomes up to 10 years after treatment<br>• Relatively large sample size, which allows for the study of multiple predictors, rare outcomes, and genetic analysis in consortia<br>• The phenotype data is of high quality and detail, including repeated measurements over time<br>• Generalisability of findings may be limited to patients in the internet psychiatry context

For peer review only

INTRODUCTION

Depression and anxiety disorders are the most common mental disorders with a lifetime prevalence of 11-14% for depression (1) and 29–34% for anxiety.(2,3) Mental disorders are the leading cause of disability worldwide, with depressive and anxiety disorders as the top contributors, together representing 11.8% of all years lived with disability (YLDs) globally among people ages 15-49.(4) Treatment options include medication and psychotherapy, where cognitive behaviour therapy (CBT) specifically has strong empirical support.(5,6) Internet-delivered CBT (ICBT) has emerged as an alternative or complement to face-to-face CBT treatment.(7–9) ICBT includes the same treatment content as face-to-face CBT, but with text-based therapy modules and remote therapist contact via typed messages, phone calls or video communication. ICBT represents a viable first-line treatment option when face-to-face treatment is not possible, available, or desired by the patient. Despite overall positive treatment outcomes, about 10-60% of patients undergoing ICBT will not respond sufficiently to treatment.(10,11) A range of different predictors for symptom change during ICBT have been suggested, including a patient's clinical history and presentation (e.g. baseline symptom severity) (10,12), the treatment process itself (13), genetic risk (14), and demographic and socioeconomic indicators.(10,12,15) Although a plethora of predictors for symptom change have been identified, findings in the field are predominantly mixed, their joint predictive power leaves room for improvement and has not resulted in widespread applicable tools for clinical decision making. One problem with these predictor findings is that they often rely on data from clinical trials, which have rather small and strictly selected samples, and consequently, are unlikely to be generalizable to new patients in real-world healthcare settings. Also, data from studies are often limited to specific diagnoses, certain types of treatments, and clinical contexts. Additionally, they rarely include multi-modal data in the same study with adequate statistical power which limits the possibility of identifying new predictors that could be helpful in guiding tailored clinical decision-making, as well as understanding mechanisms of treatment success and failure. Adaptive treatment strategies, or routine outcome monitoring, use repeated symptom measures during early treatment and constitute a promising way to reduce the risk of treatment failure for patients who are identified as at risk of negative outcomes, underscoring the clinical utility of uncovering predictive risk factors.(16,17) In summary, current predictor findings have not yet translated to a useful pre-treatment decision support tools for clinicians to identify patients at risk for negative outcomes who could instead be offered an alternative treatment.

Genetic variation explains a substantial portion of variance in depression and anxiety with an estimated twin heritability ranging from 30-60% (18,19) and a SNP (single nucleotide polymorphism) heritability ranging from 15-30%.(20–22) Depression and anxiety disorders are clearly polygenic conditions, which entails that multiple genes contribute to the shared effect.(23) Polygenicity is also expected for other complex traits, such as outcome in therapy. For several disorders, genetic variation has been associated with psychotropic drug treatment outcomes.(24–26) To this date, only one GWAS of symptom reduction in CBT has been conducted, including 2724 participants but with no genome wide significant findings, suggesting that the sample size was too small.(27)

For peer review only

MULTI-PSYCH is a longitudinal cohort of genotyped and richly phenotyped individuals with depression and anxiety disorders who have undergone highly structured treatment with internet-delivered cognitive behaviour therapy (ICBT). The aims of the project are summarised in **Figure 1**. This cohort profile paper describes the rationale, aims, methods and preliminary findings of the project.

COHORT DESCRIPTION

Participants, assessment, and setting

Participants were recruited from September 2009 through November 2019. All had a diagnosis of major depressive disorder (MDD), social anxiety disorder (SAD) or panic disorder (PD) confirmed at the Internet Psychiatry Clinic (IPSY; https://www.internetpsykiatri.se/), in Region Stockholm, Sweden.(28) Since 2007, IPSY has offered ICBT as part of the psychiatric care within the public health care system provided by Region Stockholm. The majority of patients at IPSY are self-referred and the rest referred by their general practitioner.

Each patient at IPSY initially completed an online screening procedure, including psychometric questionnaires and general questions about access to Internet, etc. Thereafter they were assessed by a clinician (psychiatrist, clinical psychologist, supervised resident), either at the clinic or via videoconferencing software. At the assessment, patients were asked whether they would like to take part in the study. Out of 5704 eligible individuals, 3298 consented to be part of the study and 2668 provided clinical data. Participation required no extra tasks except donating a blood sample for DNA extraction. Baseline demographic and clinical data were collected. Clinical diagnosis was established according to the Diagnostic and Statistical Manual of Mental Disorders (DSM-IV-TR or DSM-5(29,30)) using the clinician-administered Mini-International Neuropsychiatric Interview (MINI).(31) For enrolment the patients had to have (i) a diagnosis of MDD, SAD or PD, (ii) be able to read and write in Swedish, (iii) have computer and Internet access, (and iv) be ≥18 years old. Exclusion criteria were (v) severe MDD and/or moderate to high risk of suicide, medication (vi) medication changes less than 3 months prior to treatment start, (vii) comorbid bipolar or psychotic disorders, (viii) participation in concurrent psychotherapy, (ix) current alcohol or drug abuse/dependence, (x) communication difficulties that would impact treatment (including language difficulties), and (xi) low motivation. Enrolled patients were offered treatment which usually began the following day. See **Table 1** for participant characteristics.

The present study was approved by the Regional Ethics Board in Stockholm, Sweden (REPN: 2009/1089-31/2) and adheres to the Declaration of Helsinki.

Table 1. Sociodemographic and clinical characteristics at baseline and post treatment

	Depression (n = 1300)	Social anxiety (n = 640)	Panic disorder (n = 728)	Total (n = 2668)
Age	37.6 (11.9)	32.7 (10.3)	34.6 (10.8)	35.6 (11.4)
Sex, female	859 (66)	360 (56)	435 (60)	1654 (62)
Missing			1 (0)	1 (0)

Highest education attained				
Primary	25 (2)	14 (2)	18 (2)	57 (2)
Secondary	116 (9)	56 (9)	82 (11)	254 (10)
Higher	1158 (89)	569 (89)	626 (86)	2353 (88)
Missing	3 (0)	1 (0)	1 (0)	5 (0)
In a relationship	731 (56)	363 (57)	478 (66)	1572 (59)
Missing	3 (0)	1 (0)	1 (0)	5 (0)
Has children	603 (46)	198 (31)	301 (41)	1102 (41)
Missing	3 (0)	1 (0)	1 (0)	5 (0)
Previous suicide attempt(s)*	76 (6)	34 (5)	26 (4)	136 (6)
Missing	95 (7)	49 (8)	84 (12)	228 (9)
Previous psychiatric inpatient care*	91 (7)	27 (4)	74 (10)	192 (7)
Missing	55 (4)	30 (4)	30 (4)	115 (4)
Psychiatric comorbidity**	395 (30)	212 (33)	274 (38)	881 (33)
Psychotropic medication**	788 (61)	322 (50)	445 (61)	1555 (58)
MADRS-S/LSAS-SR/PDSS-SR pre***	22.7 (6.3)	70.6 (23.5)	11.0 (4.7)	
Missing	14 (1)	62 (10)	16 (2)	92 (3)
MADRS-S/LSAS-SR/PDSS-SR post***	13.0 (8.0)	50.1 (24)	4.98 (4.53)	
Missing	248 (19)	118 (18)	158 (21)	524 (20)

Values are decimal mean (SD) or integer count (%). *Self-reported at pre-treatment assessment **Registered at pre-treatment assessment. ***Scores only reported from scales for corresponding diagnosis. MADRS-S: Montgomery Åsberg depression rating scale self-rated, LSAS-SR: Liebowitz social anxiety scale self-rated, PDSS-SR: Panic disorder severity scale self-rated.

Internet-delivered cognitive behaviour therapy

An overview of the ICBT content is provided in **Table 2** and details can be found in.(32–34) In summary, all ICBT treatments are evidence-based protocols and was supported by a trained psychologist through written messages and homework feedback.(33–35) The ICBT treatment was module-based and self-paced, but the patients were encouraged to finish one module per week. Treatment length was set to 12 weeks and could only be extended in special cases (an early version of the social anxiety treatment was 14 weeks, but later shortened to 12 weeks). The therapist followed up on the homework and, once completed, granted access to the next module.

Table 2. Summary of content in ICBT protocols

Module/ diagnosis	Depression	Social anxiety disorder	Panic disorder

For peer review only

1	Psychoeducation about the target disorder		
2	Behavioural activation	Negative thoughts, cognitive reappraisal	Cognitive reappraisal
3	Behavioural activation	Treatment goals and behaviour experiments	Cognitive reappraisal
4	Cognitive reappraisal	Exposure and safety behaviours	Interoceptive exposure
5	Cognitive reappraisal	Challenges when working with exposure	Interoceptive exposure
6	Worry and anxiety	Communication	Agoraphobic exposure
7	Sleep problems	Expanding your behaviour repertoire	Exposure
8	Independent work with behavioural activation and cognitive reappraisal	Exposure	Combining exposure and interoceptive exposure
9	Summary		
10	Summarising treatment and relapse prevention		

Note: Most treatment components are applied throughout treatment and not only in the module where first introduced. Two of the programs have changed during the years (MDD, 2016 and SAD, 2019) but the main content remained the same.

Data sources

The MULTI-PSYCH project includes clinical, process, genetic, and register data spanning from birth to up to 10 years after the ICBT treatment. See **Figure 2** for an overview of data sources and **figure 3** for total numbers of individuals providing data from different sources. All data sources have been linked using the Swedish personal identification number assigned to each Swedish resident (35), and then replaced by an ID for which the key is kept by the coordinating register holder (the Swedish Board of Health and Welfare), providing pseudo-anonymized data to the researchers. Data processing has been performed with R version 4.1.(36)

Clinical data

Clinical data were collected in the digital treatment platform. A wide range of clinician- and self-administered rating scales were collected at screening, pre-treatment, during treatment, post-treatment and at 3-6 months follow-up after the end of treatment (details below).

Rating scales

Three disorder-specific questionnaires were used in their self-reported version as primary treatment outcomes for each diagnosis, and administered at screening, pre- and post-treatment and weekly through the treatment period. Corresponding, clinician-rated scales were used at assessment. All participants were also screened for all three diagnoses using self-report questionnaires. To assess depressive symptoms, the clinician-rated *Montgomery Åsberg Depression Rating Scale (MADRS,* score range 0-60), and MADRS self-rated (MADRS-S, score range 0-54) was used.(37,38) The *Liebowitz Social Anxiety Scale*

For peer review only

(LSAS) and the self-rated version LSAS-SR measure fear and avoidance in social situations on a Likert-type scale from 0 (representing no fear or avoidance) to 3 (indicating severe fear and avoidance), with a total score range of 0-144 points.(39) The *Panic Disorder Severity Scale (PDSS)* and the self-rated version (PDSS-SR) was used to measure the level of severity of panic disorder using seven items (with a score 0-4) addressing frequency and seriousness of various aspects of panic, anxiety, avoidance, and impairment. The total score ranges from 0 to 28.(40,41)

The following instruments are answered on one or more occasions at screening, pre-, and/or post-treatment by all or subgroups of patients, regardless of diagnosis: *Patient Health Questionnaire* (PHQ-9, depression symptoms) (42); *Social Phobia Screening* Questionnaire (SPSQ, social anxiety symptom severity) (43); *Social Phobia Inventory* (SPIN, social anxiety symptom severity) (44); *Generalized Anxiety Disorder 7-item scale* (GAD-7, symptoms of generalized anxiety) (45); *Short Health Anxiety Inventory* (SHAI, health anxiety symptoms) (46)); *Alcohol Use Disorder Identification Test* (AUDIT, screening for alcohol risk use, abuse and dependence) (47); *Drug Use Disorder Identification Test* (DUDIT, screening instrument for drug use, abuse and dependence) (48); *Insomnia severity index* (ISI, insomnia symptom severity) (49); *WHO Adult ADHD Self-Report Scale Screener v.1.1(*ASRS*,* screening for adult ADHD symptoms) (50)*; Clinical Global Impression-Severity (*CGI-S*,* general symptom severity, clinician-rated) (51)*; Clinical Global Impression-Improvement (*CGI-I*,* general improvement, clinician-rated*)* (51)*; General Self Efficacy Scale (*GSE*,* perceived general self-efficacy)(52)*: WHO Disability Assessment Schedule 2.0* (WHODAS 2.0*,* health and disability) (53)*; EuroQol-5D (*EQ-5D*,* overall health and quality of life*)* (54); *Treatment Inventory of Costs in Patients with psychiatric disorders (*TiC-P*,* healthcare consumption and productivity loss in patients with a psychiatric disorder).(55)

Process data

The treatment platform automatically recorded process data including participants' treatment behaviours, time spent in the platform or completing questionnaires, number of messages written or received, and time of day, day of week and week of year for certain activities. Process data provides detail on patients' in-treatment behaviour.

Genetic data

A total number of 3298 patients donated blood samples at the IPSY clinic. DNA was extracted for genotyping at Karolinska Institutet's biobank where samples are stored. Genotyping was performed at LIFE & BRAIN GmbH (Bonn, Germany) in three different batches. Batch 1 contained a set of 423 individuals diagnosed with PD, genotyped on an Illumina CoreExome 12v1 array. The second batch was a set of 971 individuals with MDD, genotyped on an Illumina Infinium Global Screening Array 24v1 array. The third batch was a set of 1900 individuals with either MDD, SAD or PD, genotyped on an Illumina Infinium Global Screening Array 24v2 array. All the following steps were done on batches separately to avoid batch effects, using PLINK v1.90b3n.(56) A within-cohort pruning based on genotype missingness was performed and then tested for the presence of cryptic relatedness in the data. Relatedness pruning left 3269 samples. The pre-imputation quality control involved the following, in order: removal of SNPs with call rate < 0.95; removal of samples with call rate across SNPs < 0.98; removal of samples with FHET outside of -0.2 to 0.2; removal of samples with reported/derived sex discordance; removal of samples with

For peer review only - http://bmjopen.bmj.com/site/about/guidelines.xhtml

ambiguous derived sex; removal of SNPs with a call rate < 0.98; and removal of SNPs with Hardy-Weinberg equilibrium p-value < 1e-6. The pre-imputation QC process left a total of 3251 samples. Imputation of common variant genotypes was conducted using a 1000 genomes phase 3 reference panel.(57)

MULTI-PSYCH includes SNP level data as well as aggregated polygenic risk scores (PRSs), calculated from large genome wide association studies (GWAS), were generated for the following eight traits: attention-deficit hyperactivity disorder (ADHD)(58), autism spectrum disorder (ASD)(59), bipolar disorder(60), MDD(61), schizophrenia (62), intelligence (IQ)(63), educational attainment(64), and a psychiatric cross-disorder PRS (65), using PRS-CS (66) from GWAS summary statistics and samples with European ancestry from the 1000 genomes project.(57) Additional PRSs for other relevant traits, or more powerful calculations of PRSs will be added as needed.

Register data

Variables from several Swedish registers were linked to clinical, process and genetic data for each patient. Register data will be used both as pre-treatment predictors and long-term outcomes. Below follows a summary of specific registers used, their benefits and drawbacks, and examples of key variables extracted for the project.

Medical Birth Register

Since 1973, the Medical Birth Register (MBR) records data on all pregnancies in Sweden that result in delivery and it has a 98,6 % coverage.(67) From this register patient-level data on e.g., APGAR scores, birth weight, gestational age, and perinatal complications, is used.

Total Population Register and Longitudinal integrated database for health insurance and labour market studies

The Total Population Register (TPR) managed by Statistics Sweden (Statistiska Centralbyrån, SCB) registers a multitude of data on all Swedish citizens, including immigration/emigration, civil status, family composition, and geographical location.(68) TPR has a coverage of >99 % for births and deaths, and 91-95 % for immigrations/emigrations. Data from a further processed subset of variables measured annually and collated in the *Longitudinal integrated database for health insurance and labour market studies* (known as LISA in its Swedish acronym).(69) LISA includes data on disposable income, educational attainment, country of birth and labour market marginalisation including e.g. unemployment, sickness absence and disability pension. TPR data are available from 1965 onwards and variables in LISA from 1990 and thereafter.

Education Register

The Swedish Register of Education annually collects individual-level data on educational attainment level and type for all Swedish citizens 16-74 years on January 1st each year. From this register, we used individual-level data on total score and subject grades. Some variables are available from 1973, and a majority from 1998 and onwards.(70)

National Patient Register

The National Patient Register (NPR) records all in- and out-patient visits in specialised care in Sweden to governmental and private caregiving organisations.(71) Individual-level data

For peer review only

on e.g. diagnostic codes, using the International Classification of Diseases (ICD) including psychiatric and somatic diagnoses and suicide is registered, as well as all health care interventions such as treatments and assessments with >99% coverage. Data from outpatient care is available from 2001 onwards, and from inpatient care from 1967.

VAL

VAL registers outpatient and inpatient care events in Stockholm County, to which a vast majority of MULTI-PSYCH patients belong. NPR, however, has national coverage, so VAL data has only been used for primary care events, which are lacking in NPR. This combination allows for MULTI-PSYCH to cover both primary and secondary care with high detail and completeness. VAL includes similar variables as NPR; individual-level data on diagnoses, treatments, and interventions.

Prescribed Drug Register

The Swedish Prescribed Drug Register (PDR) records all dispensations of prescribed medications at all pharmacies in the country.(72,73) Data on prescription and dispensation (medicinal outtake) dates, active substance, packaging, and dosage is registered. Data from PDR is available from July 2005.

Cause of Death Register

The Cause of Death Register (CDR) records the time of death and ICD coded cause-specific reasons for death. A great strength with the CDR is its almost perfect coverage (>99%).(74) Both time to death by any cause, and also cause-specific deaths by suicide and external deaths are available in the MULTI-PSYCH cohort.

Patient and public involvement

There was no patient or public involvement in the design or conduction of the study.

FINDINGS TO DATE

Studies have already used or are currently using the MULTI-PSYCH cohort. One study investigated the association between genetic risk scores for several psychiatric and cognitive traits to symptom reduction in ICBT in a sample of 894 patients with MDD. An association was found with the PRS for ASD. The higher the score on ASD_{PRS}, the less the depression symptoms decreased over time. The PRS for the other psychiatric and cognitive traits (including MDD) were not related to depressive symptom severity or change over time.(14) Another study used the same sample and supervised machine learning for prediction of MDD remission specifically investigating the utility of multi-modal (clinical, register, genetic) predictors available at baseline before treatment with ICBT to derive a new, multimodal classifier for prediction of MDD remission status.(75) In terms of feature engineering, a study applying longitudinal clustering of weekly symptom trajectories consisting of weekly symptom ratings will investigate if a data-driven clustering of symptoms across time during ICBT can be predictive of clinical and socioeconomic outcomes, the latter in linked registers.(76) A range of predictive studies are applied on an overlapping larger sample, using only clinical data.(77–79) In addition, MULTI-PSYCH has and will contribute data to several GWAS studies, including a GWAS meta-analysis of symptoms of depression and anxiety following CBT and found no SNPs associated with post-treatment symptoms.(27) Another GWAS, using the depression cohort from MULTI-PSYCH as controls, found evidence that individuals receiving electroconvulsive therapy for depression had more common variant risk loci than those

with mild-moderate depression, suggesting a different genetic architecture for severe cases of depression.(80) A third GWAS of the genetic architecture of PD generated highly significant PRSs and a significant genetic correlations between PD and MDD, depressive symptoms, and neuroticism.(81)

MULTI-PSYCH IN CONTEXT

The British GLAD cohort includes ~40 000 individuals with anxiety and depression.(82) Participants are recruited through advertisement and sign up to be a part of the study online, and provide genetic data, questionnaire data, and data from linked medical records. Another example is the NESDA cohort (83), which includes around 2300 individuals (plus controls) with a diagnosis of depression or anxiety. The NESDA cohort does long-term follow-up of participants for 9 years and includes clinical, etiological, and genetic data. Both cohorts include genetic data and provide a strong research infrastructure in different ways. MULTI-PSYCH contributes to the field with high quality long-term follow-up data from registers and using a strictly clinical population with richly phenotyped data throughout the lifespan.

STRENGTHS AND LIMITATIONS

The main strength of MULTI-PSYCH is the integration of clinical, genetic, and register data spanning from birth to up to 10 years after the end of treatment. The registers include healthcare data from primary and specialist care, socio-economic variables, kinship, birth, and death. Potential novel long-term predictors and outcomes can be analysed using register data, including perinatal complications, school performance, healthcare consumption, clinical diagnoses and interventions, income, social welfare, and labour market marginalisation.(84) In addition to detailed clinical information and repeated symptom measurements, data from the ICBT platform also includes detailed process data; variables specific to the online treatment process, such as patient interaction with the treatment platform, symptom questionnaires or particular words or phrases used by the patient in communication with the therapist, which may provide unique predictive value.(85) Moreover, MULTI-PSYCH sports high clinical relevance and ample opportunity for validation and implementation of findings since all included patients have been diagnosed and treated with standardised ICBT in routine internet psychiatry with nationwide coverage . MULTI-PSYCH limitations include the non-probabilistic sampling of participants and potential issues with the generalisability of the findings to other forms of psychological treatment for depression and anxiety disorders. The cohort is mainly self-referred, which means that participants may be different from the psychiatric patient population at large, for example due to higher-than-average education level and their own and the assessing clinicians' beliefs that they could benefit from a demanding self-help treatment. For some research questions, e.g., GWASs, MULTI-PSYCH has a limited number of participants highlighting the need for combining MULTI-PSYCH with other similar datasets for answering such research questions. MULTI-PSYCH is an ongoing project and collection of follow-up data from registers will be finished by 2029 but up to 3 years follow-up is already available for the bulk of included patients. Since the first patient finished treatment in 2009, register data has been recorded yearly.

COLLABORATION

For peer review only

Proposals for collaborative work should be addressed to the MULTI-PSYCH Steering Group. Data sharing requires ethical approval, confidentiality, and adherence to data handling praxis and regulations. We support requests for the reuse of data within the limits of available personnel and time resources. MULTI-PSYCH ethics approvals do not allow for public availability of data, and neither does Swedish law with respect to sensitive personal information. Meta data will be published open access as more data becomes available with time. Further details are available on the MULTI-PSYCH homepage https://ki.se/en/cns/multi-psych.

CONCLUSION

MULTI-PSYCH is a longitudinal cohort of genotyped and richly phenotyped individuals with depression and anxiety disorders who have undergone highly structured treatment with internet-delivered cognitive behaviour therapy (ICBT). It offers the potential to address clinically relevant research questions that are difficult to address in other ways. The overarching aim of MULTI-PSYCH is to advance precision psychiatry by means of deepened understanding of disorder mechanisms, to derive and validate precise and implementable predictions, and in the long run strengthen routine care of psychiatric patients to optimise healthcare resource use in the real-world setting.

FUTURE PLANS

MULTI-PSYCH is designed with collaboration in mind, including contributions to larger genetic and GWASs in consortia. Implementation of MULTI-PSYCH prediction models for post ICBT outcomes could guide tailored care with improved decision-support tools for clinicians, possibly minimising treatment failure and resource waste. Long-term prediction of register outcomes for anxiety and depression patients treated with ICBT is novel and could prove critically complementary for estimating and understanding the marginal risk for the patient over time - beyond the narrow clinical setting and time.

ACKNOWLEDGEMENTS

The authors wish to thank all patients at IPSY for their valuable contributions. The authors also want to acknowledge all clinicians and research nurse Monica Hellberg at IPSY for the invaluable contribution to data collection.

CONTRIBUTIONS

JB, JW, CR, NL, VK, and DMC designed the cohort. JB, JW, and CR drafted the manuscript. JB, JW, VK, EF, NHI, MM, CS, EAH, NL, HBD, EI, and CR contributed to the data collection. JB, JW, BR, EF, VK, and NHI processed and analysed the data. All authors interpreted the findings, critically revised the manuscript, and approved its final form and submission.

FUNDINGS

JW and CR acknowledge funding from the Söderström-König Foundation (SLS-941192 JW), FORTE (2018-00221 CR), the Swedish Research Council (2021-06377 JW; 2018-02487 CR) and the Center for innovative medicine (CIMED 96328 JW; 954440 CR). EF acknowledge funding from the L.J. Boëthius foundation and Stiftelsen Professor Bror Gadelius Minnesfond. VK acknowledge funding from the Swedish Research Council (2016-01961 VK) and the Erling-Persson Family Foundation (N/A VK).

For peer review only

CONFLICTS OF INTEREST

DMC receives royalties for contributing articles to UpToDate, Inc, outside this work. LFC receives royalties for contributing articles to UpToDate, Wolters Kluwer Health and for editorial work from Elsevier, outside the submitted work.

For peer review only

REFERENCES

1. Kessler RC, Bromet EJ. The epidemiology of depression across cultures. Annu Rev Public Health. 2013;34:119–38.

2. Kessler RC, Berglund P, Demler O, Jin R, Merikangas KR, Walters EE. Lifetime prevalence and age-of-onset distributions of DSM-IV disorders in the National Comorbidity Survey Replication. Arch Gen Psychiatry. 2005 Jun;62(6):593–602.

3. Bandelow B, Michaelis S. Epidemiology of anxiety disorders in the 21st century. Dialogues Clin Neurosci. 2015 Sep;17(3):327–35.

4. Vos T, Lim SS, Abbafati C, Abbas KM, Abbasi M, Abbasifard M, et al. Global burden of 369 diseases and injuries in 204 countries and territories, 1990–2019: a systematic analysis for the Global Burden of Disease Study 2019. Lancet. 2020 Oct 17;396(10258):1204–22.

5. Kaczkurkin AN, Foa EB. Cognitive-behavioral therapy for anxiety disorders: an update on the empirical evidence. Dialogues Clin Neurosci. 2015 Sep;17(3):337–46.

6. Hofmann SG, Asnaani A, Vonk IJJ, Sawyer AT, Fang A. The Efficacy of Cognitive Behavioral Therapy: A Review of Meta-analyses. Cognit Ther Res. 2012 Oct 1;36(5):427–40.

7. Arnberg FK, Linton SJ, Hultcrantz M, Heintz E, Jonsson U. Internet-delivered psychological treatments for mood and anxiety disorders: a systematic review of their efficacy, safety, and cost-effectiveness. PLoS One. 2014 May 20;9(5):e98118.

8. Etzelmueller A, Vis C, Karyotaki E, Baumeister H, Titov N, Berking M, et al. Effects of Internet-Based Cognitive Behavioral Therapy in Routine Care for Adults in Treatment for Depression and Anxiety: Systematic Review and Meta-Analysis. J Med Internet Res. 2020 Aug 31;22(8):e18100.

9. Andersson G, Carlbring P, Titov N, Lindefors N. Internet Interventions for Adults with Anxiety and Mood Disorders: A Narrative Umbrella Review of Recent Meta-Analyses. Can J Psychiatry. 2019 Jul;64(7):465–70.

10. Rozental A, Andersson G, Carlbring P. In the Absence of Effects: An Individual Patient Data Meta-Analysis of Non-response and Its Predictors in Internet-Based Cognitive Behavior Therapy. Front Psychol. 2019 Mar 29;10:589.

11. Young C, Campbell K, Dulong C. Internet-Delivered Cognitive Behavioural Therapy for Major Depression and Anxiety Disorders: A Review of Clinical Effectiveness. In: The Canadian Agency for Drugs and Technologies in Health (CADTH). Ottawa, CA: CADTH; 2018. p. 1–48.

12. Edmonds M, Hadjistavropoulos HD, Schneider LH, Dear BF, Titov N. Who benefits most from therapist-assisted internet-delivered cognitive behaviour therapy in clinical practice? Predictors of symptom change and dropout. J Anxiety Disord. 2018 Mar;54:24–32.

13. Forsell E, Isacsson N, Blom K, Jernelöv S, Ben Abdesslem F, Lindefors N, et al. Predicting treatment failure in regular care Internet-Delivered Cognitive Behavior Therapy for depression and anxiety using only weekly symptom measures. J Consult Clin Psychol. 2020 Apr;88(4):311–21.

14. Andersson E, Crowley JJ, Lindefors N, Ljótsson B, Hedman-Lagerlöf E, Boberg J, et al. Genetics of response to cognitive behavior therapy in adults with major depression: a preliminary report. Mol Psychiatry. 2019 Apr;24(4):484–90.

15. Karyotaki E, Ebert DD, Donkin L, Riper H, Twisk J, Burger S, et al. Do guided internet-based interventions result in clinically relevant changes for patients with depression? An individual participant data meta-analysis. Clin Psychol Rev. 2018 Jul;63:80–92.

For peer review only - http://bmjopen.bmj.com/site/about/guidelines.xhtml

16. Forsell E, Jernelöv S, Blom K, Kraepelien M, Svanborg C, Andersson G, et al. Proof of Concept for an Adaptive Treatment Strategy to Prevent Failures in Internet-Delivered CBT: A Single-Blind Randomized Clinical Trial With Insomnia Patients. American Journal of Psychiatry. 2019;176(4):315–23.
17. Lambert MJ, Whipple JL, Kleinstäuber M. Collecting and delivering progress feedback: A meta-analysis of routine outcome monitoring. Psychotherapy . 2018 Dec;55(4):520–37.
18. Polderman TJC, Benyamin B, de Leeuw CA, Sullivan PF, van Bochoven A, Visscher PM, et al. Meta-analysis of the heritability of human traits based on fifty years of twin studies. Nat Genet. 2015 Jul;47(7):702–9.
19. Sullivan PF, Neale MC, Kendler KS. Genetic epidemiology of major depression: review and meta-analysis. Am J Psychiatry. 2000 Oct;157(10):1552–62.
20. Purves KL, Coleman JRI, Rayner C, Hettema JM, Deckert J, McIntosh AM, et al. The common genetic architecture of anxiety disorders. 2017 Jan 1 [cited 2022 Sep 23]; Available from: https://www.research.ed.ac.uk/en/publications/the-common-genetic-architecture-of-anxiety-disorders
21. Meier SM, Trontti K, Purves KL, Als TD, Grove J, Laine M, et al. Genetic variants associated with anxiety and stress-related disorders: A genome-wide association study and mouse-model study. JAMA Psychiatry. 2019 Sep 1;76(9):924–32.
22. Martin N, Otowa T, Lee M, Hartman C, Oldehinkel A, Preisig M, et al. Meta-analysis of genome-wide association studies of anxiety disorders. Eur Neuropsychopharmacol. 2017;27:S501.
23. Plomin R, Haworth CMA, Davis OSP. Common disorders are quantitative traits. Nat Rev Genet. 2009 Dec;10(12):872–8.
24. Pain O, Hodgson K, Trubetskoy V, Ripke S, Marshe VS, Adams MJ, et al. Antidepressant Response in Major Depressive Disorder: A Genome-wide Association Study. medRxiv [Internet]. 2020; Available from: https://www.medrxiv.org/content/10.1101/2020.12.11.20245035v1.abstract
25. Hou L, Heilbronner U, Degenhardt F, Adli M, Akiyama K, Akula N, et al. Genetic variants associated with response to lithium treatment in bipolar disorder: a genome-wide association study. Lancet. 2016 Mar 12;387(10023):1085–93.
26. Yu H, Yan H, Wang L, Li J, Tan L, Deng W, et al. Five novel loci associated with antipsychotic treatment response in patients with schizophrenia: a genome-wide association study. Lancet Psychiatry. 2018 Apr;5(4):327–38.
27. Rayner C, Coleman JRI, Purves KL, Hodsoll J, Goldsmith K, Alpers GW, et al. A genome-wide association meta-analysis of prognostic outcomes following cognitive behavioural therapy in individuals with anxiety and depressive disorders. Transl Psychiatry. 2019 May 23;9(1):150.
28. Titov N, Dear B, Nielssen O, Staples L, Hadjistavropoulos H, Nugent M, et al. ICBT in routine care: A descriptive analysis of successful clinics in five countries. Internet Interv. 2018 Sep;13:108–15.
29. American Psychiatric Association. Diagnostic and Statistical Manual of Mental Disorders 5 (5th ed). 2013.
30. American Psychiatric Association. Diagnostic and statistical manual of mental disorders : DSM-IV-TR. Washington, DC: American Psychiatric Association; 2000.
31. Sheehan DV, Lecrubier Y, Sheehan KH, Amorim P, Janavs J, Weiller E, et al. The Mini-International Neuropsychiatric Interview (M.I.N.I.): the development and validation of a structured diagnostic psychiatric interview for DSM-IV and ICD-10. J Clin Psychiatry. 1998;59 Suppl 20:22-33;quiz 34-57.

For peer review only

32. Hedman E, Andersson G, Ljótsson B, Andersson E, Rück C, Mörtberg E, et al. Internet-based cognitive behavior therapy vs. cognitive behavioral group therapy for social anxiety disorder: a randomized controlled non-inferiority trial. PLoS One. 2011 Mar 25;6(3):e18001.
33. Bergström J, Andersson G, Ljótsson B, Rück C, Andréewitch S, Karlsson A, et al. Internet-versus group-administered cognitive behaviour therapy for panic disorder in a psychiatric setting: a randomised trial. BMC Psychiatry. 2010 Jul 2;10(1):54.
34. Andersson G, Bergström J, Holländare F, Carlbring P, Kaldo V, Ekselius L. Internet-based self-help for depression: randomised controlled trial. Br J Psychiatry. 2005 Nov;187:456–61.
35. Ludvigsson JF, Otterblad-Olausson P, Pettersson BU, Ekbom A. The Swedish personal identity number: possibilities and pitfalls in healthcare and medical research. Eur J Epidemiol. 2009 Jun 6;24(11):659–67.
36. Team, R Core, Others. R: A language and environment for statistical computing. 2013; Available from: http://www.R-project.org/
37. Montgomery SA, Asberg M. A new depression scale designed to be sensitive to change. Br J Psychiatry. 1979 Apr;134:382–9.
38. Svanborg P, Asberg M. A comparison between the Beck Depression Inventory (BDI) and the self-rating version of the Montgomery Asberg Depression Rating Scale (MADRS). J Affect Disord. 2001 May;64(2–3):203–16.
39. Fresco DM, Coles ME, Heimberg RG, Liebowitz MR, Hami S, Stein MB, et al. The Liebowitz Social Anxiety Scale: a comparison of the psychometric properties of self-report and clinician-administered formats. Psychol Med. 2001 Aug;31(6):1025–35.
40. Shear MK, Rucci P, Williams J, Frank E, Grochocinski V, Vander Bilt J, et al. Reliability and validity of the Panic Disorder Severity Scale: replication and extension. J Psychiatr Res. 2001 Sep;35(5):293–6.
41. Shear MK, Brown TA, Barlow DH, Money R, Sholomskas DE, Woods SW, et al. Multicenter collaborative panic disorder severity scale. Am J Psychiatry. 1997 Nov;154(11):1571–5.
42. Kroenke K, Spitzer RL, Williams JB. The PHQ-9: validity of a brief depression severity measure. J Gen Intern Med. 2001 Sep;16(9):606–13.
43. Furmark T, Tillfors M, Everz P, Marteinsdottir I, Gefvert O, Fredrikson M. Social phobia in the general population: prevalence and sociodemographic profile. Soc Psychiatry Psychiatr Epidemiol. 1999 Aug;34(8):416–24.
44. Connor KM, Davidson JR, Churchill LE, Sherwood A, Foa E, Weisler RH. Psychometric properties of the Social Phobia Inventory (SPIN). New self-rating scale. Br J Psychiatry. 2000 Apr;176:379–86.
45. Spitzer RL, Kroenke K, Williams JBW, Löwe B. A brief measure for assessing generalized anxiety disorder: the GAD-7. Arch Intern Med. 2006 May 22;166(10):1092–7.
46. Salkovskis PM, Rimes KA, Warwick HMC, Clark DM. The Health Anxiety Inventory: development and validation of scales for the measurement of health anxiety and hypochondriasis. Psychol Med. 2002 Jul;32(5):843–53.
47. Bohn MJ, Babor TF, Kranzler HR. The Alcohol Use Disorders Identification Test (AUDIT): validation of a screening instrument for use in medical settings. J Stud Alcohol. 1995 Jul;56(4):423–32.
48. Berman AH, Bergman H, Palmstierna T, Schlyter F. Evaluation of the Drug Use Disorders Identification Test (DUDIT) in criminal justice and detoxification settings and in a Swedish population sample. Eur Addict Res. 2005;11(1):22–31.
49. Bastien CH, Vallières A, Morin CM. Validation of the Insomnia Severity Index as an outcome measure for insomnia research. Sleep Med. 2001 Jul;2(4):297–307.

For peer review only

50. Kessler RC, Adler L, Ames M, Demler O, Faraone S, Hiripi E, et al. The World Health Organization Adult ADHD Self-Report Scale (ASRS): a short screening scale for use in the general population. Psychol Med. 2005 Feb;35(2):245–56.
51. Guy W. ECDEU Assessment Manual for Psychopharmacology. U.S. Department of Health, Education, and Welfare, Public Health Service, Alcohol, Drug Abuse, and Mental Health Administration, National Institute of Mental Health, Psychopharmacology Research Branch, Division of Extramural Research Programs; 1976. 603 p.
52. Sherer M, Maddux JE, Mercandante B, Prentice-Dunn S, Jacobs B, Rogers RW. The Self-Efficacy Scale: Construction and Validation. Psychol Rep. 1982 Oct 1;51(2):663–71.
53. Ustün TB, World Health Organization. Measuring health and disability : manual for WHO Disability Assessment Schedule WHODAS 2.0. Geneva: World Health Organization; 2010.
54. Rabin R, de Charro F. EQ-5D: a measure of health status from the EuroQol Group. Ann Med. 2001 Jul;33(5):337–43.
55. Bouwmans C, De Jong K, Timman R, Zijlstra-Vlasveld M, Van der Feltz-Cornelis C, Tan Swan S, et al. Feasibility, reliability and validity of a questionnaire on healthcare consumption and productivity loss in patients with a psychiatric disorder (TiC-P). BMC Health Serv Res. 2013 Jun 15;13:217.
56. Purcell S, Neale B, Todd-Brown K, Thomas L, Ferreira MAR, Bender D, et al. PLINK: a tool set for whole-genome association and population-based linkage analyses. Am J Hum Genet. 2007 Sep;81(3):559–75.
57. 1000 Genomes Project Consortium, Auton A, Brooks LD, Durbin RM, Garrison EP, Kang HM, et al. A global reference for human genetic variation. Nature. 2015 Oct 1;526(7571):68–74.
58. Demontis D, Walters RK, Martin J, Mattheisen M, Als TD, Agerbo E, et al. Discovery of the first genome-wide significant risk loci for attention deficit/hyperactivity disorder. Nat Genet. 2019 Jan;51(1):63–75.
59. Autism Spectrum Disorders Working Group of The Psychiatric Genomics Consortium. Meta-analysis of GWAS of over 16,000 individuals with autism spectrum disorder highlights a novel locus at 10q24.32 and a significant overlap with schizophrenia. Mol Autism. 2017 May 22;8:21.
60. Stahl EA, Breen G, Forstner AJ, McQuillin A, Ripke S, Trubetskoy V, et al. Genome-wide association study identifies 30 loci associated with bipolar disorder. Nat Genet. 2019 May;51(5):793–803.
61. Wray NR, Ripke S, Mattheisen M, Trzaskowski M, Byrne EM, Abdellaoui A, et al. Genome-wide association analyses identify 44 risk variants and refine the genetic architecture of major depression. Nat Genet. 2018 May;50(5):668–81.
62. Schizophrenia Working Group of the Psychiatric Genomics Consortium. Biological insights from 108 schizophrenia-associated genetic loci. Nature. 2014 Jul 24;511(7510):421–7.
63. Savage JE, Jansen PR, Stringer S, Watanabe K, Bryois J, de Leeuw CA, et al. Genome-wide association meta-analysis in 269,867 individuals identifies new genetic and functional links to intelligence. Nat Genet. 2018 Jul;50(7):912–9.
64. Okbay A, Beauchamp JP, Fontana MA, Lee JJ, Pers TH, Rietveld CA, et al. Genome-wide association study identifies 74 loci associated with educational attainment. Nature. 2016 May 26;533(7604):539–42.
65. Cross-Disorder Group of the Psychiatric Genomics Consortium. Electronic address: plee0@mgh.harvard.edu, Cross-Disorder Group of the Psychiatric Genomics Consortium.

For peer review only

Genomic Relationships, Novel Loci, and Pleiotropic Mechanisms across Eight Psychiatric Disorders. Cell. 2019 Dec 12;179(7):1469-1482.e11.
66. Ge T, Chen C-Y, Ni Y, Feng Y-CA, Smoller JW. Polygenic prediction via Bayesian regression and continuous shrinkage priors. Nat Commun. 2019 Apr 16;10(1):1776.
67. Centre for Epidemiology. The Swedish Medical Birth Register: summary of content and quality: 2003 [Internet]. [cited 2022 Sep 15]. Available from: https://www.socialstyrelsen.se/globalassets/sharepoint-dokument/artikelkatalog/ovrigt/2003-112-3_20031123.pdf
68. Ludvigsson JF, Almqvist C, Bonamy A-KE, Ljung R, Michaëlsson K, Neovius M, et al. Registers of the Swedish total population and their use in medical research. Eur J Epidemiol. 2016 Feb;31(2):125–36.
69. Ludvigsson JF, Svedberg P, Olén O, Bruze G, Neovius M. The longitudinal integrated database for health insurance and labour market studies (LISA) and its use in medical research. Eur J Epidemiol. 2019 Apr;34(4):423–37.
70. Statistiska centralbyrån SCB. Befolkningens utbildning (Utbildningsregistret, UREG) [Internet]. 2011. Available from: https://www.scb.se/contentassets/776fd7a4ac2f4574ae86e4a3b5b7595e/uf0506_do_2011.pdf
71. Ludvigsson JF, Andersson E, Ekbom A, Feychting M, Kim J-L, Reuterwall C, et al. External review and validation of the Swedish national inpatient register. BMC Public Health. 2011 Jun 9;11:450.
72. Wettermark B, Hammar N, Fored CM, Leimanis A, Otterblad Olausson P, Bergman U, et al. The new Swedish Prescribed Drug Register--opportunities for pharmacoepidemiological research and experience from the first six months. Pharmacoepidemiol Drug Saf. 2007 Jul;16(7):726–35.
73. Wallerstedt SM, Wettermark B, Hoffmann M. The First Decade with the Swedish Prescribed Drug Register - A Systematic Review of the Output in the Scientific Literature. Basic Clin Pharmacol Toxicol. 2016 Nov;119(5):464–9.
74. Brooke HL, Talbäck M, Hörnblad J, Johansson LA, Ludvigsson JF, Druid H, et al. The Swedish cause of death register. Eur J Epidemiol. 2017 Sep;32(9):765–73.
75. Wallert J, Boberg J, Kaldo V, Mataix-Cols D, Flygare O. Predicting remission after internet-delivered psychotherapy in patients with depression using machine learning and multi-modal data. medRxiv [Internet]. 2021; Available from: https://www.medrxiv.org/content/10.1101/2021.04.30.21256367v1.abstract
76. Wallert, J, Boberg, J, Kravchenko, O, Kaldo, V, Mataix-Cols, D, Crowley, J, Rück, C. Polygenic Risk Scores, Data-Driven Longitudinal Clusters of Weekly Symptom Trajectories, and Post Psychotherapy Remission in Major Depressive Disorder. World Congress of Psychiatric Genetics; 2022 Sep 15; Florence, Italy.
77. Gogoulou E, Boman M, Ben Abdesslem F, Hentati Isacsson N, Kaldo V, Sahlgren M. Predicting Treatment Outcome from Patient Texts:The Case of Internet-Based Cognitive Behavioural Therapy. In: Proceedings of the 16th Conference of the European Chapter of the Association for Computational Linguistics: Main Volume. Online: Association for Computational Linguistics; 2021. p. 575–80.
78. Boman M, Ben Abdesslem F, Forsell E, Gillblad D, Görnerup O, Isacsson N, et al. Learning machines in Internet-delivered psychological treatment. Progress in Artificial Intelligence. 2019 Dec 1;8(4):475–85.
79. Schibbye P, Ghaderi A, Ljótsson B, Hedman E, Lindefors N, Rück C, et al. Using early change to predict outcome in cognitive behaviour therapy: exploring timeframe, calculation method, and differences of disorder-specific versus general measures. PLoS One. 2014 Jun 24;9(6):e100614.

For peer review only

80. Clements CC, Karlsson R, Lu Y, Juréus A, Rück C, Andersson E, et al. Genome-wide association study of patients with a severe major depressive episode treated with electroconvulsive therapy. Mol Psychiatry [Internet]. 2021 Jan 22; Available from: https://psycnet.apa.org/fulltext/2021-11534-001.pdf
81. Forstner AJ, Awasthi S, Wolf C, Maron E, Erhardt A, Czamara D, et al. Genome-wide association study of panic disorder reveals genetic overlap with neuroticism and depression. Mol Psychiatry [Internet]. 2019 Nov 11; Available from: http://dx.doi.org/10.1038/s41380-019-0590-2
82. Davies MR, Kalsi G, Armour C, Jones IR, McIntosh AM, Smith DJ, et al. The Genetic Links to Anxiety and Depression (GLAD) Study: Online recruitment into the largest recontactable study of depression and anxiety. Behav Res Ther. 2019 Dec;123:103503.
83. Penninx BWJH, Eikelenboom M, Giltay EJ, van Hemert AM, Riese H, Schoevers RA, et al. Cohort profile of the longitudinal Netherlands Study of Depression and Anxiety (NESDA) on etiology, course and consequences of depressive and anxiety disorders. J Affect Disord. 2021 May 15;287:69–77.
84. Maret-Ouda J, Tao W, Wahlin K, Lagergren J. Nordic registry-based cohort studies: Possibilities and pitfalls when combining Nordic registry data. Scand J Public Health. 2017 Jul;45(17_suppl):14–9.
85. Wallert J, Gustafson E, Held C, Madison G, Norlund F, von Essen L, et al. Predicting Adherence to Internet-Delivered Psychotherapy for Symptoms of Depression and Anxiety After Myocardial Infarction: Machine Learning Insights From the U-CARE Heart Randomized Controlled Trial. J Med Internet Res. 2018 Oct 10;20(10):e10754.

For peer review only

Figure legends

Figure 1. MULTI-PSYCH data structure and research aims. ICBT=Internet-based cognitive behaviour therapy, SNP=Single nucleotide polymorphism, GWAS=Genome wide association study

Figure 2. Data sources. An overview of data sources and time periods. R=Register data, MBR=Medical birth register, UREG=Swedish register of education, NPR=National patient register, PDR=Prescribed drug register, LISA=Longitudinal integrated database for health insurance and labour market studies, VAL=Stockholm County health care register, TPR=Total population register, IPSY=Internet psychiatry clinic, CDR=Causes of death register.

Figure 3. Available data. The number of individuals providing data from different data sources.

For peer review only

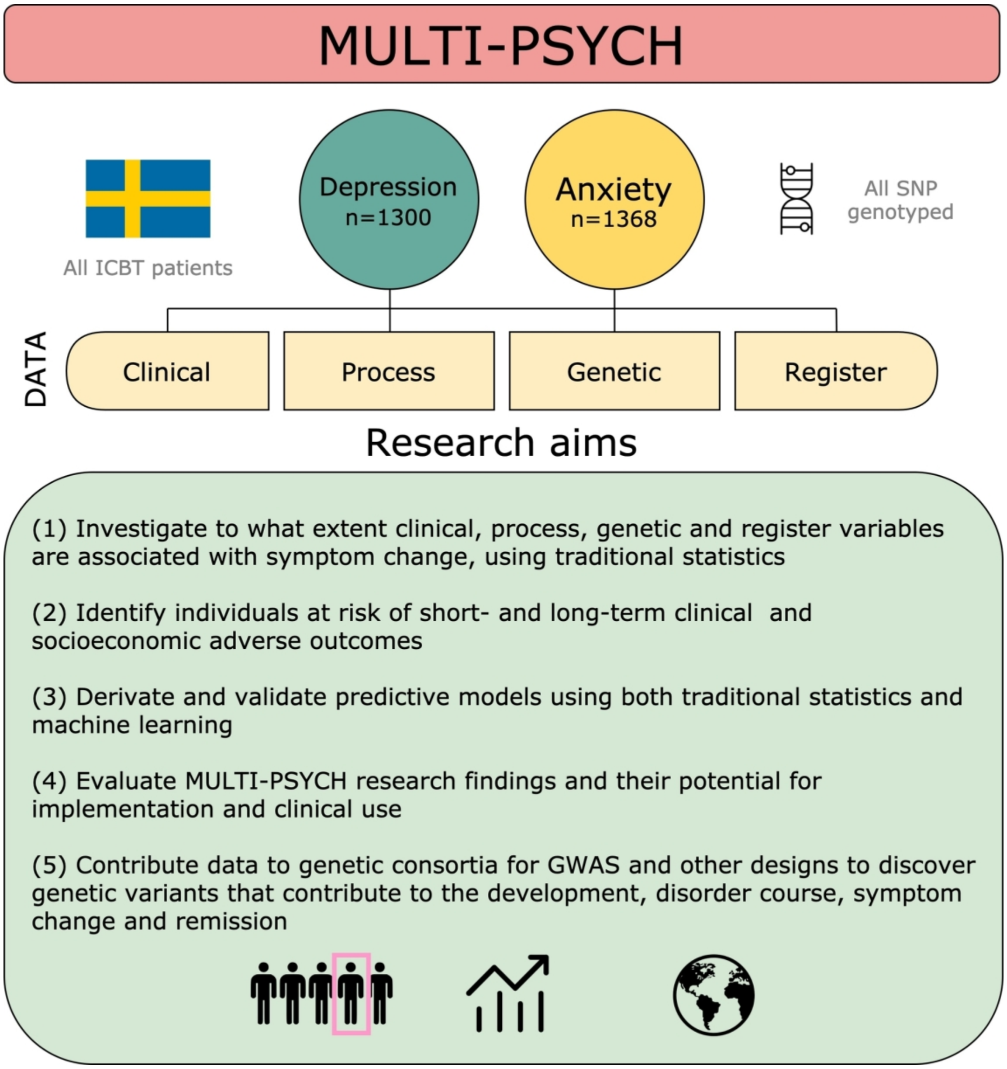

Figure 1. MULTI-PSYCH data structure and research aims. ICBT=Internet-based cognitive behaviour therapy, SNP=Single nucleotide polymorphism, GWAS=Genome wide association study

163x172mm (300 x 300 DPI)

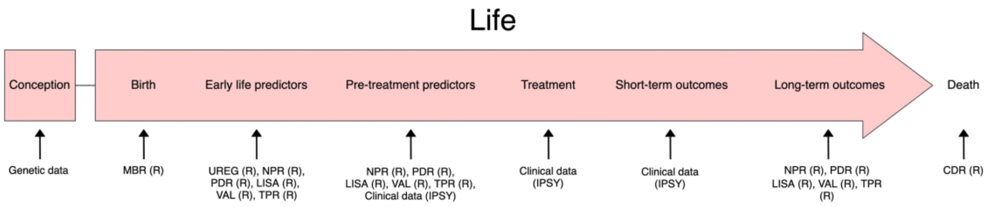

Figure 2. Data sources. An overview of data sources and time periods. R=Register data, MBR=Medical birth register, UREG=Swedish register of education, NPR=National patient register, PDR=Prescribed drug register, LISA=Longitudinal integrated database for health insurance and labour market studies, VAL=Stockholm County health care register, TPR=Total population register, IPSY=Internet psychiatry clinic, CDR=Causes of death register.

337x69mm (300 x 300 DPI)

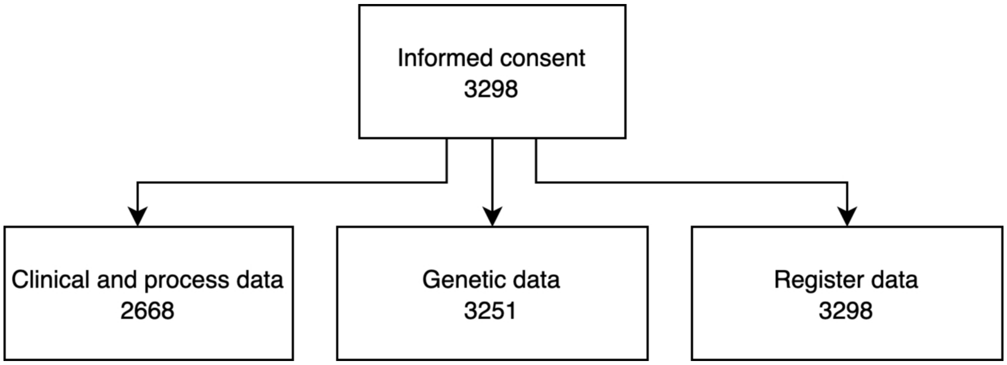

Figure 3. Available data. The number of individuals providing data from different data sources.

114x40mm (300 x 300 DPI)

BMJ Open

A Swedish multimodal cohort of patients with anxiety or depression treated with internet-delivered psychotherapy (MULTI-PSYCH)

Journal:	*BMJ Open*
Manuscript ID	bmjopen-2022-069427.R1
Article Type:	Cohort profile
Date Submitted by the Author:	14-Jul-2023
Complete List of Authors:	Boberg, Julia; Centre for Psychiatry Research, Department of Clinical Neuroscience, Karolinska Institutet, & Stockholm Health Care Services, Region Stockholm<br>Kaldo, V; Centre for Psychiatry Research, Department of Clinical Neuroscience, Karolinska Institutet, & Stockholm Health Care Services, Region Stockholm; Linnaeus University Faculty of Health and Life Sciences, Department of Psychology<br>Mataix-Cols, David; Centre for Psychiatry Research, Department of Clinical Neuroscience, Karolinska Institutet, & Stockholm Health Care Services, Region Stockholm<br>Crowley, James; The University of North Carolina at Chapel Hill, Department of Genetics; Centre for Psychiatry Research, Department of Clinical Neuroscience, Karolinska Institutet, & Stockholm Health Care Services, Region Stockholm<br>Roelstraete, Bjorn; Karolinska Institutet, Department of Medical Epidemiology and Biostatistics<br>Halvorsen, Matthew; The University of North Carolina at Chapel Hill, Department of Genetics<br>Forsell, Erik; Centre for Psychiatry Research, Department of Clinical Neuroscience, Karolinska Institutet, & Stockholm Health Care Services, Region Stockholm<br>Isacsson, Nils; Centre for Psychiatry Research, Department of Clinical Neuroscience, Karolinska Institutet, & Stockholm Health Care Services, Region Stockholm<br>Sullivan, Patrick F.; Karolinska Institutet, Department of Medical Epidemiology and Biostatistics; The University of North Carolina at Chapel Hill, Department of Genetics<br>Svanborg, Cecilia; Centre for Psychiatry Research, Department of Clinical Neuroscience, Karolinska Institutet, & Stockholm Health Care Services, Region Stockholm<br>Andersson, Evelyn; Centre for Psychiatry Research, Department of Clinical Neuroscience, Karolinska Institutet, & Stockholm Health Care Services, Region Stockholm<br>Lindefors, Nils; Centre for Psychiatry Research, Department of Clinical Neuroscience, Karolinska Institutet, & Stockholm Health Care Services, Region Stockholm<br>Kravchenko, Olly; Centre for Psychiatry Research, Department of Clinical Neuroscience, Karolinska Institutet, & Stockholm Health Care Services, Region Stockholm

	Mattheisen, Manuel; Aarhus Universitet, Department of Biomedicine; Centre for Psychiatry Research, Department of Clinical Neuroscience, Karolinska Institutet, & Stockholm Health Care Services, Region Stockholm<br>Danielsdottir, Hilda; Centre for Psychiatry Research, Department of Clinical Neuroscience, Karolinska Institutet, & Stockholm Health Care Services, Region Stockholm<br>Ivanova, Ekaterina; Centre for Psychiatry Research, Department of Clinical Neuroscience, Karolinska Institutet, & Stockholm Health Care Services, Region Stockholm<br>Boman, Magnus; Karolinska Institutet, Department of Learning, Informatics, Management and Ethics, ; KTH Royal Institute of Technology, Department of Computer and Software Systems, School of EECS<br>Fernández de la Cruz, Lorena; Centre for Psychiatry Research, Department of Clinical Neuroscience, Karolinska Institutet, & Stockholm Health Care Services, Region Stockholm<br>Wallert, John; Centre for Psychiatry Research, Department of Clinical Neuroscience, Karolinska Institutet, & Stockholm Health Care Services, Region Stockholm<br>Rück, Christian; Centre for Psychiatry Research, Department of Clinical Neuroscience, Karolinska Institutet, & Stockholm Health Care Services, Region Stockholm
**Primary Subject Heading**:	Genetics and genomics
Secondary Subject Heading:	Genetics and genomics
Keywords:	Anxiety disorders < PSYCHIATRY, Depression & mood disorders < PSYCHIATRY, GENETICS, EPIDEMIOLOGY

SCHOLARONE™
Manuscripts

I, the Submitting Author has the right to grant and does grant on behalf of all authors of the Work (as defined in the below author licence), an exclusive licence and/or a non-exclusive licence for contributions from authors who are: i) UK Crown employees; ii) where BMJ has agreed a CC-BY licence shall apply, and/or iii) in accordance with the terms applicable for US Federal Government officers or employees acting as part of their official duties; on a worldwide, perpetual, irrevocable, royalty-free basis to BMJ Publishing Group Ltd ("BMJ") its licensees and where the relevant Journal is co-owned by BMJ to the co-owners of the Journal, to publish the Work in this journal and any other BMJ products and to exploit all rights, as set out in our licence.

The Submitting Author accepts and understands that any supply made under these terms is made by BMJ to the Submitting Author unless you are acting as an employee on behalf of your employer or a postgraduate student of an affiliated institution which is paying any applicable article publishing charge ("APC") for Open Access articles. Where the Submitting Author wishes to make the Work available on an Open Access basis (and intends to pay the relevant APC), the terms of reuse of such Open Access shall be governed by a Creative Commons licence – details of these licences and which Creative Commons licence will apply to this Work are set out in our licence referred to above.

Other than as permitted in any relevant BMJ Author's Self Archiving Policies, I confirm this Work has not been accepted for publication elsewhere, is not being considered for publication elsewhere and does not duplicate material already published. I confirm all authors consent to publication of this Work and authorise the granting of this licence.

For peer review only

A Swedish multimodal cohort of patients with anxiety or depression treated with internet-delivered psychotherapy (MULTI-PSYCH)

Julia Boberg[1,] Viktor Kaldo[1,2], David Mataix-Cols[1], James J Crowley[1,4,5], Bjorn Roelstraete[3], Matthew W Halvorsen[4], Erik Forsell[1], Nils Hentati Isacsson[1], Patrick F Sullivan[3,4,5], Cecilia Svanborg[1], Evelyn Andersson[1], Nils Lindefors[1], Olly Kravchenko[1], Manuel Mattheisen[1,8,9], Hilda Björk Daníelsdóttir[1], Ekaterina Ivanova[1], Magnus Boman[6,7], Lorena Fernández de la Cruz[1], John Wallert[1*], Christian Rück[1*]

*shared last author

Corresponding author: Julia Boberg, julia.boberg@ki.se, M46, Psychiatry Southwest, Karolinska University Hospital, SE-141 86 Huddinge, Sweden.

[1]Centre for Psychiatry Research, Department of Clinical Neuroscience, Karolinska Institutet, & Stockholm Health Care Services, Region Stockholm, Stockholm, Sweden

[2]Department of Psychology, Faculty of Health and Life Sciences, Linnaeus University, Växjö, Sweden.

[3]Department of Medical Epidemiology and Biostatistics, Karolinska Institutet, Stockholm, Sweden.

[4]Department of Genetics, University of North Carolina, Chapel Hill, North Carolina.

[5]Department of Psychiatry, University of North Carolina, Chapel Hill, North Carolina.

[6]Department of Learning, Informatics, Management and Ethics, Karolinska Institutet, Stockholm, Sweden.

[7]Department of Computer and Software Systems, School of EECS, KTH, Stockholm, Sweden.

[8]Department of Biomedicine, Aarhus University, Aarhus, Denmark.

[9]Department of Psychiatry, Psychosomatics and Psychotherapy, University of Würzburg, Würzburg, Germany.

Word count: 3796

For peer review only

Abstract

Purpose: Depression and anxiety afflict millions worldwide causing considerable disability. MULTI-PSYCH is a longitudinal cohort of genotyped and phenotyped individuals with depression or anxiety disorders who have undergone highly structured internet-based cognitive behaviour therapy (ICBT). The overarching purpose of MULTI-PSYCH is to improve risk stratification, outcome prediction, and secondary preventive interventions. MULTI-PSYCH is a precision medicine initiative that combines clinical, genetic, and nationwide register data.

Participants: MULTI-PSYCH includes 2668 clinically well-characterised adults with major depressive disorder (MDD) (n=1300), social anxiety disorder (n=640) or panic disorder (n=728) assessed before, during, and after 12 weeks of ICBT at the Internet psychiatry clinic in Stockholm, Sweden. All patients have been blood sampled and genotyped. Clinical and genetic data has been linked to several Swedish registers containing a wide range of variables from patient birth up to 10 years after the end of ICBT. These variable types include perinatal complications, school grades, psychiatric and somatic comorbidity, dispensed medications, medical interventions and diagnoses, health care and social benefits, demographics, income and more. Long-term follow-up data will be collected through 2029.

Findings to date: Initial uses of MULTI-PSYCH include the discovery of an association between PRS for autism spectrum disorder and response to ICBT, the development of a machine learning model for baseline prediction of remission status after ICBT in MDD, and data contributions to genome wide association studies (GWAS) for ICBT outcome. Other projects have been launched or are in the planning phase.

Future plans: The MULTI-PSYCH cohort provides a unique infrastructure to study not only predictors or short-term treatment outcomes, but also longer-term medical and socioeconomic outcomes in patients treated with ICBT for depression or anxiety. MULTI-PSYCH is well-positioned for research collaboration.

Strengths and limitations of this study
• MULTI-PSYCH is a unique integration of clinical, genetic, and register data for psychiatric patients treated with ICBT for depression or anxiety<br>• Long-term follow-up across different data sources give opportunity to study multiple relevant outcomes up to a decade after ICBT completion<br>• Relatively large sample size facilitating the study of many predictors, rare outcomes, and consortia genetic analysis<br>• Phenotype data are granular and of high quality, including weekly symptom ratings at item level<br>• Generalisability of findings is limited to adult patients with depression or anxiety treated with ICBT

Data availability statement

For peer review only

Data are available on reasonable request. Data sharing requires ethical approval, confidentiality, and adherence to data handling praxis and regulations. We support requests for the reuse of data within the limits of available personnel and time resources and encourage collaboration with other researchers. MULTI-PSYCH ethical approvals do not allow for public availability of data, and neither does Swedish law with respect to sensitive personal information.

For peer review only

INTRODUCTION

Depression and anxiety disorders are the most common mental disorders with a lifetime prevalence of 11-14% (1) and 29–34% (2,3), respectively. Suffering from a mental disorder is a leading cause of disability worldwide, and depressive and anxiety disorders are the most frequent, together representing 11.8% of all years lived with disability (YLDs) globally among people ages 15-49 (4). Treatment options include medication and psychotherapy, and particularly cognitive behaviour therapy (CBT) has strong empirical support (5,6). Over the last decades, Internet-delivered CBT (ICBT) has been validated as an alternative or complement to face-to-face CBT treatment (7–10). ICBT includes the same treatment content as face-to-face CBT, but with text-based therapy modules and remote therapist contact via typed messages, phone calls or video communication. ICBT represents a viable first-line treatment option when face-to-face treatment is not possible, available, or desired by the patient. Despite overall positive treatment outcomes, about 10-60% of patients undergoing ICBT will not respond sufficiently to treatment, and some even deteriorate (11,12). If we could predict, before the start of ICBT, which patients will not be helped by treatment, more potent tailored intervention and also more effective use of scarce psychiatric resources could be achieved.

Towards that overarching goal, a range of predictors for symptom change during ICBT have been suggested. These include baseline symptom severity (11,13), the treatment process (14), demographic and socioeconomic variables (11,13,15), genetic risk (16), and more. Although a plethora of predictors for symptom change have been identified, findings in the field are predominantly mixed and their joint predictive power has not resulted in widespread applicable prediction tools for clinical decision making. One problem with prior findings is that they often rely on data from clinical trials, which have rather small and strictly selected samples. Consequently, generalisability of such findings to new patients in real-world healthcare settings is limited. Also, these studies are often focused on specific diagnoses, certain types of treatments, and clinical contexts. Additionally, they rarely include multi-modal data which limits the possibility of identifying new predictors that could be helpful in guiding tailored clinical decision-making, as well as understanding mechanisms of treatment success and failure. Adaptive treatment strategies, or routine outcome monitoring, use repeated symptom measures during early treatment and constitute a promising way to reduce the risk of treatment failure for patients who are identified as at risk of negative outcomes, underscoring the clinical utility of uncovering predictive risk factors (17,18). In summary, current predictor findings have not yet translated to a useful pre-treatment decision support tools for clinicians to identify patients at risk for negative outcomes who could instead be offered an alternative treatment.

Genetic variation explains a substantial portion of variance in depression and anxiety disorders with an estimated twin heritability ranging from 30-60% (19,20) and a SNP (single nucleotide polymorphism) heritability ranging from 15-30% (21–23). Depression and anxiety disorders are clearly polygenic conditions, meaning that many genes contribute to the effect (24). Such polygenicity is also expected for other complex traits, including

For peer review only

therapy outcome. For several disorders, genetic variation has been associated with psychotropic drug treatment outcomes (25–27). To this date, only one GWAS of symptom change in CBT has been conducted, including 2724 participants, but with no genome wide significant findings, suggesting a too small sample size (28).

MULTI-PSYCH is a longitudinal cohort of genotyped and phenotyped individuals with depression or anxiety disorders who have undergone highly structured treatment with ICBT. MULTI-PSYCH provides excellent opportunities for collaboration with other researchers. Aims of the project are summarised in **Figure 1**. This cohort profile paper describes the rationale, aims, methods, and preliminary findings of the project.

COHORT DESCRIPTION

Participants, assessment, and setting

The present study was approved by the Regional Ethics Board in Stockholm, Sweden (REPN: 2009/1089-31/2) and adheres to the Declaration of Helsinki.

Participants were recruited from September 2009 through November 2019. All had a diagnosis of either major depressive disorder (MDD), social anxiety disorder or panic disorder confirmed at the Internet Psychiatry Clinic (IPSY; https://www.internetpsykiatri.se/), in Region Stockholm, Sweden (29). Since 2007, IPSY has offered ICBT as part of the psychiatric care within the public health care system provided by Region Stockholm. The majority of patients at IPSY are self-referred and the rest referred by their general practitioner.

Each patient at IPSY initially completed an online screening procedure, including psychometric questionnaires and general questions about Internet access, and more. Thereafter they were assessed by a clinician (psychiatrist/clinical psychologist/supervised resident), either at the clinic or via video. Patients were asked at assessment whether they would like to take part in the study. Out of 5704 eligible individuals, 3298 consented to be part of the study and 2668 provided clinical data. Participation required no extra tasks except donating a blood sample for DNA extraction. Baseline demographic and clinical data were collected. Formal diagnosis was established according to the Diagnostic and Statistical Manual of Mental Disorders (DSM-IV-TR or DSM-5 (30,31)) using the clinician-administered Mini-International Neuropsychiatric Interview (MINI) (32). For enrolment in treatment a patients had to (i) have a diagnosis of MDD, social anxiety disorder or panic disorder, (ii) be able to read and write in Swedish, (iii) have computer and Internet access, (and iv) be ≥18 years old. Exclusion criteria were (v) severe MDD and/or moderate to high risk of suicide, (vi) comorbid bipolar or psychotic disorder, (vii) participation in concurrent psychotherapy, (viii) current alcohol or drug abuse/dependence, (ix) communication difficulties that would impact treatment (including language difficulties), and (x) low motivation. Enrolled patients were offered treatment which usually began the following day. See **Table 1** for participant characteristics.

Table 1. Sociodemographic and clinical characteristics at baseline and post treatment

	MDD (n = 1300)	Social anxiety disorder (n = 640)	Panic disorder (n = 728)	Total (n = 2668)

Age	37.6 (11.9)	32.7 (10.3)	34.6 (10.8)	35.6 (11.4)
Sex, female	859 (66)	360 (56)	435 (60)	1654 (62)
Missing			1 (0)	1 (0)
Highest education attained				
Primary	25 (2)	14 (2)	18 (2)	57 (2)
Secondary	116 (9)	56 (9)	82 (11)	254 (10)
Higher	1158 (89)	569 (89)	626 (86)	2353 (88)
Missing	3 (0)	1 (0)	1 (0)	5 (0)
In a relationship	731 (56)	363 (57)	478 (66)	1572 (59)
Missing	3 (0)	1 (0)	1 (0)	5 (0)
Has children	603 (46)	198 (31)	301 (41)	1102 (41)
Missing	3 (0)	1 (0)	1 (0)	5 (0)
Previous suicide attempt(s)*	76 (6)	34 (5)	26 (4)	136 (6)
Missing	95 (7)	49 (8)	84 (12)	228 (9)
Previous psychiatric inpatient care*	91 (7)	27 (4)	74 (10)	192 (7)
Missing	55 (4)	30 (4)	30 (4)	115 (4)
Psychiatric comorbidity**	395 (30)	212 (33)	274 (38)	881 (33)
Psychotropic medication**	788 (61)	322 (50)	445 (61)	1555 (58)
MADRS-S/LSAS-SR/PDSS-SR pre***	22.7 (6.3)	70.6 (23.5)	11.0 (4.7)	
Missing	14 (1)	62 (10)	16 (2)	92 (3)
MADRS-S/LSAS-SR/PDSS-SR post***	13.0 (8.0)	50.1 (24)	4.98 (4.53)	
Missing	248 (19)	118 (18)	158 (21)	524 (20)

Values are decimal mean (SD) or integer count (%). *Self-reported at pre-treatment assessment **Registered at pre-treatment assessment. *** Scores are reported from the scale corresponding to each diagnosis . MADRS-S: Montgomery Åsberg depression rating scale self-rated, LSAS-SR: Liebowitz social anxiety scale self-rated, PDSS-SR: Panic disorder severity scale self-rated.

For peer review only

Internet-delivered cognitive behaviour therapy

An overview of the ICBT content is provided in **Table 2** and details can be found in (33–35). In summary, all ICBT treatment protocols were evidence-based and included therapist support by a trained psychologist through written messages and homework feedback (33–35). Treatment was module-based and self-paced, but the patients were encouraged to finish one module per week. Treatment length was set to 12 weeks (an early version of the social anxiety treatment was 14 weeks, but later shortened to 12). The therapist followed up on homework and, once completed, granted access to the next module.

Table 2. Summary of content in ICBT protocols

Module/ diagnosis	Depression	Social anxiety disorder	Panic disorder
1	Psychoeducation about the target disorder		
2	Behavioural activation	Negative thoughts, cognitive reappraisal	Cognitive reappraisal
3	Behavioural activation	Treatment goals and behaviour experiments	Cognitive reappraisal
4	Cognitive reappraisal	Exposure and safety behaviours	Interoceptive exposure
5	Cognitive reappraisal	Challenges when working with exposure	Interoceptive exposure
6	Worry and anxiety	Communication	Agoraphobic exposure
7	Sleep problems	Expanding your behaviour repertoire	Exposure
8	Independent work with behavioural activation and cognitive reappraisal	Exposure	Combining exposure and interoceptive exposure
9	Summary		
10	Summarising treatment and relapse prevention		

Note: Most treatment components are applied throughout treatment and not only in the module where first introduced. Two of the programs have changed during the years (MDD, 2016 and social anxiety disorder, 2019) but the main content remained the same.

Data sources

The MULTI-PSYCH project includes clinical, process, genetic, and register data spanning from birth to up to 10 years after the ICBT treatment. See **Figure 2** for an overview of data sources and **figure 3** for the total number of individuals providing data from different sources. All data sources have been linked using the Swedish personal identification number assigned to each Swedish resident (36), and then replaced by an ID for which the key is kept by the coordinating register holder (Socialstyrelsen, the Swedish Board of Health and Welfare), providing pseudo-anonymized data to the researchers. Data processing has been performed with R version 4.1 (37).

Clinical data

Clinical data were collected in the digital treatment platform. A wide range of clinician- and self-administered rating scales were administrated at screening, pre-treatment, during treatment, post-treatment, and at 3-6 months after end of treatment (details below).

Rating scales

Three disorder-specific questionnaires were used in their self-reported versions as primary treatment outcome for each diagnosis, and administered at screening, pre-, post-, and weekly throughout the treatment period. Corresponding, clinician-rated scales were used at pre-treatment assessment. To assess depressive symptoms, the clinician-rated *Montgomery Åsberg Depression Rating Scale (*MADRS, score range 0-60), and MADRS self-rated (MADRS-S, score range 0-54) were used (38,39). The *Liebowitz Social Anxiety Scale*

For peer review only - http://bmjopen.bmj.com/site/about/guidelines.xhtml

(LSAS) and the self-rated version LSAS-SR which estimates fear and avoidance in social situations, with a total score range of 0-144 points (40). The *Panic Disorder Severity Scale* (PDSS) and the self-rated version (PDSS-SR) were used to measure the level of severity of panic disorder addressing frequency and severity level of various aspects of panic, anxiety, avoidance, and impairment. The total score ranges from 0 to 28 (41,42).

The following instruments were answered on one or more occasions at screening, pre-, and/or post-treatment by all or subgroups of patients: *Patient Health Questionnaire* (PHQ-9, depression symptoms) (43); *Social Phobia Screening* Questionnaire (SPSQ, social anxiety symptom severity) (44); *Social Phobia Inventory* (SPIN, social anxiety symptom severity) (45); *Generalized Anxiety Disorder 7-item scale* (GAD-7, symptoms of generalized anxiety) (46); *Short Health Anxiety Inventory* (SHAI, health anxiety symptoms) (47)); *Alcohol Use Disorder Identification Test* (AUDIT, screening for alcohol risk use, abuse, and dependence) (48); *Drug Use Disorder Identification Test* (DUDIT, screening instrument for drug use, abuse, and dependence) (49); *Insomnia severity index* (ISI, insomnia symptom severity) (50); *WHO Adult ADHD Self-Report Scale Screener v.1.1*(ASRS, screening for adult ADHD symptoms) (51); *Clinical Global Impression-Severity* (CGI-S, general symptom severity, clinician-rated) (52); *Clinical Global Impression-Improvement* (CGI-I, general improvement, clinician-rated) (52); *General Self Efficacy Scale* (GSE, perceived general self-efficacy)(53): *WHO Disability Assessment Schedule 2.0* (WHODAS 2.0, health and disability) (54); *EuroQol-5D* (EQ-5D, overall health and quality of life) (55); *Treatment Inventory of Costs in Patients with psychiatric disorders* (TiC-P, healthcare consumption and productivity loss in patients with a psychiatric disorder) (56).

Process data

The treatment platform automatically recorded process data, including participants' treatment behaviours, time spent in the platform, time spent completing questionnaires, number of messages written or received, and time of day/day of week/week of year for treatment activities.

Genetic data

A total number of 3298 patients donated blood samples at IPSY. DNA was extracted at Karolinska Institutet's biobank where samples are stored. Genotyping was performed at LIFE & BRAIN GmbH (Bonn, Germany) in three different batches. Batch 1 contained a set of 423 individuals diagnosed with panic disorder, genotyped on an Illumina CoreExome 12v1 array. The second batch was a set of 971 individuals with MDD, genotyped on an Illumina Infinium Global Screening Array 24v1 array. The third batch was a set of 1900 individuals with either MDD, social anxiety disorder or panic disorder, genotyped on an Illumina Infinium Global Screening Array 24v2 array. All the following steps were done on batches separately to avoid batch effects, using PLINK v1.90b3n (57). A within-cohort pruning based on genotype missingness was performed and then tested for the presence of cryptic relatedness in the data. Relatedness pruning left 3269 samples. The pre-imputation quality control involved the following, in order: removal of SNPs with call rate < 0.95; removal of samples with call rate across SNPs < 0.98; removal of samples with FHET outside of -0.2 to 0.2; removal of samples with reported/derived sex discordance; removal of samples with ambiguous derived sex; removal of SNPs with a call rate < 0.98; and removal of SNPs with Hardy-Weinberg equilibrium p-value < 1e-6. The pre-imputation

For peer review only - http://bmjopen.bmj.com/site/about/guidelines.xhtml

QC process left a total of 3251 samples. Imputation of common variant genotypes was conducted using a 1000 genomes phase 3 reference panel (58).

MULTI-PSYCH includes SNP level data as well as polygenic risk scores (PRSs), calculated from large genome wide association studies (GWAS). PRSs were generated for the following eight traits: attention-deficit hyperactivity disorder (59), autism spectrum disorder (60), bipolar disorder (61), MDD (62), schizophrenia (63), intelligence/IQ (64), educational attainment (65), and a psychiatric cross-disorder PRS (66), using PRS-CS (67) from GWAS summary statistics and samples with European ancestry from the 1000 genomes project (58). Additional PRSs for other relevant traits, or more powerful calculations of PRSs will be added in the future as these become available.

Register data

Variables from several Swedish registers were linked for each patient. Register data will be used both as pre-treatment predictors and long-term outcomes. MULTI-PSYCH will further gather data for each year until 10 years after treatment start from all relevant registers. Registers are usually updated on a yearly basis, and the cohort database will be updated accordingly with additional data until 2029. Long-term follow-up data includes information from all registers except the Medical Birth Register and the Swedish Register of Education (see figure 2). This long-term follow-up allows us to investigate the patients' course after ICBT in a both longer and broader sense than mere symptom change close to end of ICBT treatment. The long-term follow-up includes relevant health and socioeconomic factors such as labour market marginalisation, disposable income, health interventions, and prescribed drugs. Below follows a summary of specific registers used, their benefits and drawbacks, and examples of key variables used in MULTI-PSYCH.

Medical Birth Register

Since 1973, the Medical Birth Register records data on all pregnancies in Sweden that result in delivery, with a 98,6 % coverage (68). From this register patient-level data on e.g., APGAR scores, birth weight, gestational age, and perinatal complications, were used.

Total Population Register and Longitudinal integrated database for health insurance and labour market studies

The Total Population Register managed by Statistics Sweden (Statistiska Centralbyrån, SCB) registers detailed information on all Swedish citizens, including immigration/emigration, civil status, family composition, and geographical location (69). Total Population Register has a coverage of >99% for births and deaths, and 91-95 % for immigrations/emigrations. Data from a further processed subset of variables measured annually and collated in the *Longitudinal integrated database for health insurance and labour market studies* (known as LISA in its Swedish acronym) were used (70). LISA includes data on disposable income, educational attainment, country of birth and labour market marginalisation, based on days of unemployment, sickness absence, and disability pension. Total Population Register data are available from 1965 and onwards and variables in LISA from 1990 and thereafter.

Education Register

For peer review only

The Swedish Register of Education annually collects individual-level data on educational attainment level and type for all Swedish citizens 16-74 years on January 1st each year. From this register, we used data on total score and subject grades. Some variables are available from 1973, and a majority from 1998 and onwards (71).

National Patient Register

The National Patient Register records all in- and out-patient visits in specialised care at both governmental and private caregiving organisations (72). Data on diagnostic codes, using the International Classification of Diseases (ICD) including psychiatric and somatic diagnoses and suicide is registered, as well as all health care interventions with >99% coverage. Data from and from inpatient care is available from 1967 and outpatient care is from 2001 onwards.

VAL

VAL registers outpatient and inpatient care events in Stockholm County, to which a majority of MULTI-PSYCH patients belong. Since the National Patient Register has national coverage, VAL data was only used for primary care events, which are lacking in the National Patient Register. This combination allows for MULTI-PSYCH to cover both primary and secondary care with high detail and completeness. VAL includes similar variables as the National Patient Register; individual-level data on diagnoses, treatments, and interventions.

Prescribed Drug Register

The Swedish Prescribed Drug Register records all dispensations of prescribed medications at all pharmacies in the country (73,74). Data on prescription and dispensation (medicinal outtake) dates, active substance, packaging, and dosage is registered. Data from the Swedish Prescribed Drug Register records is available from July 2005.

Cause of Death Register

The Cause of Death Register records the time of death and ICD coded cause-specific reasons for death. A great strength with the Cause of Death Register is its almost perfect coverage (>99%) (75). Both time to death by any cause, and cause of death, including suicide, are available in the MULTI-PSYCH cohort.

Patient and public involvement

There was no patient or public involvement in the design or conduction of the study.

FINDINGS TO DATE

Researchers have already used or are currently using the MULTI-PSYCH cohort. One study investigated the association between genetic risk scores for several psychiatric and cognitive traits to symptom reduction in ICBT in a sample of 894 patients with MDD. An association was found with the PRS for autism; the higher the score on autism$_{PRS}$, the less the depression symptoms decreased over time. The PRS for the other psychiatric and cognitive traits (including MDD) were not related to depressive symptom severity or change over time (16). Another study used the same sample applying supervised machine learning for prediction of MDD remission, with a particular focus on the potential utility of multi-modal (clinical, genetic, register) predictors available at baseline before treatment with ICBT. The study derived a new, multimodal classifier for prediction of MDD remission status (76). In terms of feature engineering, a study applying longitudinal

For peer review only - http://bmjopen.bmj.com/site/about/guidelines.xhtml

clustering of weekly symptom trajectories consisting of weekly symptom ratings will investigate if a data-driven clustering of symptoms across time during ICBT can be predictive of clinical and socioeconomic outcomes, the latter in linked registers (77). A range of predictive studies are applied on an overlapping larger sample, using only clinical data (78–80). In addition, MULTI-PSYCH has and will continue to contribute data to GWAS, including a GWAS meta-analysis of symptoms of depression or anxiety following CBT. The study found no SNPs associated with post-treatment symptoms (28). Another GWAS, using the depression cohort from MULTI-PSYCH as controls, found evidence that individuals receiving electroconvulsive therapy for depression had more common variant risk loci than those with mild-moderate depression, suggesting a different genetic architecture for severe cases of depression (81). A third GWAS of the genetic architecture of panic disorder generated highly significant PRSs and a significant genetic correlations between panic disorder and MDD, depressive symptoms, and neuroticism (82).

MULTI-PSYCH IN CONTEXT

The British GLAD cohort includes ~40 000 individuals with anxiety and depression (83). Participants are recruited through advertisement and sign up to be a part of the study online, and provide genetic data, questionnaire data, and data from linked medical records. Another example is the NESDA cohort (84), which includes around 2300 individuals (plus controls) with a diagnosis of depression or anxiety. The NESDA cohort does long-term follow-up of participants for 9 years and includes clinical, etiological, and genetic data. Both cohorts include genetic data and provide a strong research infrastructure in different ways. MULTI-PSYCH contributes to the field with high quality long-term follow-up data from registers and using a strictly clinical population with richly phenotyped data throughout the lifespan. The aim of MULTI-PSYCH is to collaborate with other cohorts to increase sample sizes and generalisability of findings.

STRENGTHS AND LIMITATIONS

The main strength of MULTI-PSYCH is the integration of clinical, genetic, and register data spanning from birth to up to 10 years after the end of treatment. It includes high-quality data from multiple nationwide registers, leveraging data from primary and specialist care, socioeconomic and demographics, kinship, birth, death, and more. Potential novel long-term predictors and outcomes can be analysed using register data, including perinatal complications, school performance, healthcare consumption, clinical diagnoses and interventions, income, social welfare, and labour market marginalisation (85). In addition to detailed clinical information and repeated symptom measurements, data from the ICBT platform also includes detailed process data; variables specific to the online treatment process, such as patient interaction with the treatment platform, symptom questionnaires or particular words or phrases used by the patient in communication with the therapist, which may provide unique insights (86). Moreover, MULTI-PSYCH holds clinical relevance and opportunity for validation and implementation of findings since all included patients have been diagnosed and treated with standardised ICBT in routine care. Limitations include the non-probabilistic sampling of participants and potential issues with the generalisability of the findings to other forms of psychological treatment for depression or anxiety disorders. The cohort is mainly self-referred, which means that participants may be different from the psychiatric patient population at large, e.g. having a higher-than-average education level plus their own and the assessing clinicians' beliefs that they could benefit from a

For peer review only

demanding self-help ICBT treatment. On the other hand, MULTI-PSYCH is highly generalisable to the context of ICBT treatment for anxiety and depression disorders, both with respect to mode of referral and inclusion/exclusion criteria. For some research questions, e.g., GWASs, MULTI-PSYCH has a limited number of participants highlighting the need for combining data with other similar datasets for answering such research questions. MULTI-PSYCH is an ongoing project and collection of follow-up data from registers will be finished by 2029 but up to 3 years follow-up is already available for the bulk of included patients. Since the first patient finished treatment in 2009, register data has been recorded yearly.

COLLABORATION

Proposals for collaborative work should be addressed to the MULTI-PSYCH Steering Group. Data sharing requires ethical approval, confidentiality, and adherence to data handling praxis and regulations. Meta data will be published open access as more data becomes available with time. Further details are available on the MULTI-PSYCH homepage https://ki.se/en/cns/multi-psych.

CONCLUSION

MULTI-PSYCH is a longitudinal cohort of genotyped and richly phenotyped individuals with depression or anxiety disorders who have undergone highly structured treatment with ICBT. It offers potential to investigate clinically relevant research questions that are difficult to address in other ways. The overarching aim of MULTI-PSYCH is to advance precision psychiatry by means of deepened understanding of disorder mechanisms, to derive and validate precise and implementable predictions, and in the long run strengthen routine care of psychiatric patients to optimise healthcare resource use in the real-world setting.

FUTURE PLANS

MULTI-PSYCH is designed with collaboration in mind, including contributions to larger genetic studies and GWASs in consortia. Implementation of MULTI-PSYCH prediction models for post ICBT outcomes could guide tailored care with improved decision-support tools for clinicians, possibly minimising treatment failure and resource waste. Long-term prediction of register outcomes for anxiety and depression patients treated with ICBT is novel and could prove critically complementary for estimating and understanding the marginal risk for the patient over time - beyond the narrow clinical setting and time.

ACKNOWLEDGEMENTS

The authors wish to thank all patients at IPSY for their valuable contributions. The authors also want to acknowledge all clinicians and research nurse Monica Hellberg at IPSY for the invaluable contribution to data collection.

CONTRIBUTIONS

JB, JW, CR, NL, VK, and DMC designed the cohort. JB, JW, and CR drafted the manuscript. JB, JW, VK, EF, NHI, MM, CS, EAH, NL, HBD, EI, and CR contributed to the data collection. JB, JW, BR, EF, VK, and NHI processed and analysed the data. JB, VK, DMC, JC, BR, MH, EF, NHI, PFS, CS, EHA, NL, OK, MM, HBD, EI, MB, LFC, JW,

and CR interpreted the findings, critically revised the manuscript, and approved its final form and submission.

FUNDINGS

JW and CR acknowledge funding from the Söderström-König Foundation (SLS-941192 JW), FORTE (2018-00221 CR), the Swedish Research Council (2021-06377 JW; 2018-02487 CR) and the Centre for innovative medicine (CIMED 96328 JW; 954440 CR). EF acknowledge funding from the L.J. Boëthius foundation and Stiftelsen Professor Bror Gadelius Minnesfond. VK acknowledge funding from the Swedish Research Council (2016-01961 VK) and the Erling-Persson Family Foundation (N/A VK).

CONFLICTS OF INTEREST

DMC receives royalties for contributing articles to UpToDate, Inc, outside this work. LFC receives royalties for contributing articles to UpToDate, Wolters Kluwer Health and for editorial work from Elsevier, outside the submitted work.

For peer review only

REFERENCES

1. Kessler RC, Bromet EJ. The epidemiology of depression across cultures. Annu Rev Public Health. 2013;34:119–38.

2. Kessler RC, Berglund P, Demler O, Jin R, Merikangas KR, Walters EE. Lifetime prevalence and age-of-onset distributions of DSM-IV disorders in the National Comorbidity Survey Replication. Arch Gen Psychiatry. 2005 Jun;62(6):593–602.

3. Bandelow B, Michaelis S. Epidemiology of anxiety disorders in the 21st century. Dialogues Clin Neurosci. 2015 Sep;17(3):327–35.

4. Vos T, Lim SS, Abbafati C, Abbas KM, Abbasi M, Abbasifard M, et al. Global burden of 369 diseases and injuries in 204 countries and territories, 1990–2019: a systematic analysis for the Global Burden of Disease Study 2019. Lancet. 2020 Oct 17;396(10258):1204–22.

5. Kaczkurkin AN, Foa EB. Cognitive-behavioral therapy for anxiety disorders: an update on the empirical evidence. Dialogues Clin Neurosci. 2015 Sep;17(3):337–46.

6. Hofmann SG, Asnaani A, Vonk IJJ, Sawyer AT, Fang A. The Efficacy of Cognitive Behavioral Therapy: A Review of Meta-analyses. Cognit Ther Res. 2012 Oct 1;36(5):427–40.

7. Arnberg FK, Linton SJ, Hultcrantz M, Heintz E, Jonsson U. Internet-delivered psychological treatments for mood and anxiety disorders: a systematic review of their efficacy, safety, and cost-effectiveness. PLoS One. 2014 May 20;9(5):e98118.

8. Etzelmueller A, Vis C, Karyotaki E, Baumeister H, Titov N, Berking M, et al. Effects of Internet-Based Cognitive Behavioral Therapy in Routine Care for Adults in Treatment for Depression and Anxiety: Systematic Review and Meta-Analysis. J Med Internet Res. 2020 Aug 31;22(8):e18100.

9. Andersson G, Carlbring P, Titov N, Lindefors N. Internet Interventions for Adults with Anxiety and Mood Disorders: A Narrative Umbrella Review of Recent Meta-Analyses. Can J Psychiatry. 2019 Jul;64(7):465–70.

10. Hedman-Lagerlöf E, Carlbring P, Svärdman F, Riper H, Cuijpers P, Andersson G. Therapist-supported Internet-based cognitive behaviour therapy yields similar effects as face-to-face therapy for psychiatric and somatic disorders: an updated systematic review and meta-analysis. World Psychiatry. 2023 Jun;22(2):305–14.

11. Rozental A, Andersson G, Carlbring P. In the Absence of Effects: An Individual Patient Data Meta-Analysis of Non-response and Its Predictors in Internet-Based Cognitive Behavior Therapy. Front Psychol. 2019 Mar 29;10:589.

12. Young C, Campbell K, Dulong C. Internet-Delivered Cognitive Behavioural Therapy for Major Depression and Anxiety Disorders: A Review of Clinical Effectiveness. In: The Canadian Agency for Drugs and Technologies in Health (CADTH). Ottawa, CA: CADTH; 2018. p. 1–48.

13. Edmonds M, Hadjistavropoulos HD, Schneider LH, Dear BF, Titov N. Who benefits most from therapist-assisted internet-delivered cognitive behaviour therapy in clinical practice? Predictors of symptom change and dropout. J Anxiety Disord. 2018 Mar;54:24–32.

14. Forsell E, Isacsson N, Blom K, Jernelöv S, Ben Abdesslem F, Lindefors N, et al. Predicting treatment failure in regular care Internet-Delivered Cognitive Behavior Therapy for depression and anxiety using only weekly symptom measures. J Consult Clin Psychol. 2020 Apr;88(4):311–21.

15. Karyotaki E, Ebert DD, Donkin L, Riper H, Twisk J, Burger S, et al. Do guided internet-based interventions result in clinically relevant changes for patients with

For peer review only

depression? An individual participant data meta-analysis. Clin Psychol Rev. 2018 Jul;63:80–92.
16. Andersson E, Crowley JJ, Lindefors N, Ljótsson B, Hedman-Lagerlöf E, Boberg J, et al. Genetics of response to cognitive behavior therapy in adults with major depression: a preliminary report. Mol Psychiatry. 2019 Apr;24(4):484–90.
17. Forsell E, Jernelöv S, Blom K, Kraepelien M, Svanborg C, Andersson G, et al. Proof of Concept for an Adaptive Treatment Strategy to Prevent Failures in Internet-Delivered CBT: A Single-Blind Randomized Clinical Trial With Insomnia Patients. American Journal of Psychiatry. 2019;176(4):315–23.
18. Lambert MJ, Whipple JL, Kleinstäuber M. Collecting and delivering progress feedback: A meta-analysis of routine outcome monitoring. Psychotherapy . 2018 Dec;55(4):520–37.
19. Polderman TJC, Benyamin B, de Leeuw CA, Sullivan PF, van Bochoven A, Visscher PM, et al. Meta-analysis of the heritability of human traits based on fifty years of twin studies. Nat Genet. 2015 Jul;47(7):702–9.
20. Sullivan PF, Neale MC, Kendler KS. Genetic epidemiology of major depression: review and meta-analysis. Am J Psychiatry. 2000 Oct;157(10):1552–62.
21. Purves KL, Coleman JRI, Rayner C, Hettema JM, Deckert J, McIntosh AM, et al. The common genetic architecture of anxiety disorders. 2017 Jan 1 [cited 2022 Sep 23]; Available from: https://www.research.ed.ac.uk/en/publications/the-common-genetic-architecture-of-anxiety-disorders
22. Meier SM, Trontti K, Purves KL, Als TD, Grove J, Laine M, et al. Genetic variants associated with anxiety and stress-related disorders: A genome-wide association study and mouse-model study. JAMA Psychiatry. 2019 Sep 1;76(9):924–32.
23. Martin N, Otowa T, Lee M, Hartman C, Oldehinkel A, Preisig M, et al. Meta-analysis of genome-wide association studies of anxiety disorders. Eur Neuropsychopharmacol. 2017;27:S501.
24. Plomin R, Haworth CMA, Davis OSP. Common disorders are quantitative traits. Nat Rev Genet. 2009 Dec;10(12):872–8.
25. Pain O, Hodgson K, Trubetskoy V, Ripke S, Marshe VS, Adams MJ, et al. Antidepressant Response in Major Depressive Disorder: A Genome-wide Association Study. medRxiv [Internet]. 2020; Available from: https://www.medrxiv.org/content/10.1101/2020.12.11.20245035v1.abstract
26. Hou L, Heilbronner U, Degenhardt F, Adli M, Akiyama K, Akula N, et al. Genetic variants associated with response to lithium treatment in bipolar disorder: a genome-wide association study. Lancet. 2016 Mar 12;387(10023):1085–93.
27. Yu H, Yan H, Wang L, Li J, Tan L, Deng W, et al. Five novel loci associated with antipsychotic treatment response in patients with schizophrenia: a genome-wide association study. Lancet Psychiatry. 2018 Apr;5(4):327–38.
28. Rayner C, Coleman JRI, Purves KL, Hodsoll J, Goldsmith K, Alpers GW, et al. A genome-wide association meta-analysis of prognostic outcomes following cognitive behavioural therapy in individuals with anxiety and depressive disorders. Transl Psychiatry. 2019 May 23;9(1):150.
29. Titov N, Dear B, Nielssen O, Staples L, Hadjistavropoulos H, Nugent M, et al. ICBT in routine care: A descriptive analysis of successful clinics in five countries. Internet Interv. 2018 Sep;13:108–15.
30. American Psychiatric Association. Diagnostic and Statistical Manual of Mental Disorders 5 (5th ed). 2013.
31. American Psychiatric Association. Diagnostic and statistical manual of mental disorders : DSM-IV-TR. Washington, DC: American Psychiatric Association; 2000.

For peer review only

32. Sheehan DV, Lecrubier Y, Sheehan KH, Amorim P, Janavs J, Weiller E, et al. The Mini-International Neuropsychiatric Interview (M.I.N.I.): the development and validation of a structured diagnostic psychiatric interview for DSM-IV and ICD-10. J Clin Psychiatry. 1998;59 Suppl 20:22-33;quiz 34-57.

33. Hedman E, Andersson G, Ljótsson B, Andersson E, Rück C, Mörtberg E, et al. Internet-based cognitive behavior therapy vs. cognitive behavioral group therapy for social anxiety disorder: a randomized controlled non-inferiority trial. PLoS One. 2011 Mar 25;6(3):e18001.

34. Bergström J, Andersson G, Ljótsson B, Rück C, Andréewitch S, Karlsson A, et al. Internet-versus group-administered cognitive behaviour therapy for panic disorder in a psychiatric setting: a randomised trial. BMC Psychiatry. 2010 Jul 2;10(1):54.

35. Andersson G, Bergström J, Holländare F, Carlbring P, Kaldo V, Ekselius L. Internet-based self-help for depression: randomised controlled trial. Br J Psychiatry. 2005 Nov;187:456–61.

36. Ludvigsson JF, Otterblad-Olausson P, Pettersson BU, Ekbom A. The Swedish personal identity number: possibilities and pitfalls in healthcare and medical research. Eur J Epidemiol. 2009 Jun 6;24(11):659–67.

37. Team, R Core, Others. R: A language and environment for statistical computing. 2013; Available from: http://www.R-project.org/

38. Montgomery SA, Asberg M. A new depression scale designed to be sensitive to change. Br J Psychiatry. 1979 Apr;134:382–9.

39. Svanborg P, Asberg M. A comparison between the Beck Depression Inventory (BDI) and the self-rating version of the Montgomery Asberg Depression Rating Scale (MADRS). J Affect Disord. 2001 May;64(2–3):203–16.

40. Fresco DM, Coles ME, Heimberg RG, Liebowitz MR, Hami S, Stein MB, et al. The Liebowitz Social Anxiety Scale: a comparison of the psychometric properties of self-report and clinician-administered formats. Psychol Med. 2001 Aug;31(6):1025–35.

41. Shear MK, Rucci P, Williams J, Frank E, Grochocinski V, Vander Bilt J, et al. Reliability and validity of the Panic Disorder Severity Scale: replication and extension. J Psychiatr Res. 2001 Sep;35(5):293–6.

42. Shear MK, Brown TA, Barlow DH, Money R, Sholomskas DE, Woods SW, et al. Multicenter collaborative panic disorder severity scale. Am J Psychiatry. 1997 Nov;154(11):1571–5.

43. Kroenke K, Spitzer RL, Williams JB. The PHQ-9: validity of a brief depression severity measure. J Gen Intern Med. 2001 Sep;16(9):606–13.

44. Furmark T, Tillfors M, Everz P, Marteinsdottir I, Gefvert O, Fredrikson M. Social phobia in the general population: prevalence and sociodemographic profile. Soc Psychiatry Psychiatr Epidemiol. 1999 Aug;34(8):416–24.

45. Connor KM, Davidson JR, Churchill LE, Sherwood A, Foa E, Weisler RH. Psychometric properties of the Social Phobia Inventory (SPIN). New self-rating scale. Br J Psychiatry. 2000 Apr;176:379–86.

46. Spitzer RL, Kroenke K, Williams JBW, Löwe B. A brief measure for assessing generalized anxiety disorder: the GAD-7. Arch Intern Med. 2006 May 22;166(10):1092–7.

47. Salkovskis PM, Rimes KA, Warwick HMC, Clark DM. The Health Anxiety Inventory: development and validation of scales for the measurement of health anxiety and hypochondriasis. Psychol Med. 2002 Jul;32(5):843–53.

48. Bohn MJ, Babor TF, Kranzler HR. The Alcohol Use Disorders Identification Test (AUDIT): validation of a screening instrument for use in medical settings. J Stud Alcohol. 1995 Jul;56(4):423–32.

For peer review only

49. Berman AH, Bergman H, Palmstierna T, Schlyter F. Evaluation of the Drug Use Disorders Identification Test (DUDIT) in criminal justice and detoxification settings and in a Swedish population sample. Eur Addict Res. 2005;11(1):22–31.
50. Bastien CH, Vallières A, Morin CM. Validation of the Insomnia Severity Index as an outcome measure for insomnia research. Sleep Med. 2001 Jul;2(4):297–307.
51. Kessler RC, Adler L, Ames M, Demler O, Faraone S, Hiripi E, et al. The World Health Organization Adult ADHD Self-Report Scale (ASRS): a short screening scale for use in the general population. Psychol Med. 2005 Feb;35(2):245–56.
52. Guy W. ECDEU Assessment Manual for Psychopharmacology. U.S. Department of Health, Education, and Welfare, Public Health Service, Alcohol, Drug Abuse, and Mental Health Administration, National Institute of Mental Health, Psychopharmacology Research Branch, Division of Extramural Research Programs; 1976. 603 p.
53. Sherer M, Maddux JE, Mercandante B, Prentice-Dunn S, Jacobs B, Rogers RW. The Self-Efficacy Scale: Construction and Validation. Psychol Rep. 1982 Oct 1;51(2):663–71.
54. Ustün TB, World Health Organization. Measuring health and disability : manual for WHO Disability Assessment Schedule WHODAS 2.0. Geneva: World Health Organization; 2010.
55. Rabin R, de Charro F. EQ-5D: a measure of health status from the EuroQol Group. Ann Med. 2001 Jul;33(5):337–43.
56. Bouwmans C, De Jong K, Timman R, Zijlstra-Vlasveld M, Van der Feltz-Cornelis C, Tan Swan S, et al. Feasibility, reliability and validity of a questionnaire on healthcare consumption and productivity loss in patients with a psychiatric disorder (TiC-P). BMC Health Serv Res. 2013 Jun 15;13:217.
57. Purcell S, Neale B, Todd-Brown K, Thomas L, Ferreira MAR, Bender D, et al. PLINK: a tool set for whole-genome association and population-based linkage analyses. Am J Hum Genet. 2007 Sep;81(3):559–75.
58. 1000 Genomes Project Consortium, Auton A, Brooks LD, Durbin RM, Garrison EP, Kang HM, et al. A global reference for human genetic variation. Nature. 2015 Oct 1;526(7571):68–74.
59. Demontis D, Walters RK, Martin J, Mattheisen M, Als TD, Agerbo E, et al. Discovery of the first genome-wide significant risk loci for attention deficit/hyperactivity disorder. Nat Genet. 2019 Jan;51(1):63–75.
60. Autism Spectrum Disorders Working Group of The Psychiatric Genomics Consortium. Meta-analysis of GWAS of over 16,000 individuals with autism spectrum disorder highlights a novel locus at 10q24.32 and a significant overlap with schizophrenia. Mol Autism. 2017 May 22;8:21.
61. Stahl EA, Breen G, Forstner AJ, McQuillin A, Ripke S, Trubetskoy V, et al. Genome-wide association study identifies 30 loci associated with bipolar disorder. Nat Genet. 2019 May;51(5):793–803.
62. Wray NR, Ripke S, Mattheisen M, Trzaskowski M, Byrne EM, Abdellaoui A, et al. Genome-wide association analyses identify 44 risk variants and refine the genetic architecture of major depression. Nat Genet. 2018 May;50(5):668–81.
63. Schizophrenia Working Group of the Psychiatric Genomics Consortium. Biological insights from 108 schizophrenia-associated genetic loci. Nature. 2014 Jul 24;511(7510):421–7.
64. Savage JE, Jansen PR, Stringer S, Watanabe K, Bryois J, de Leeuw CA, et al. Genome-wide association meta-analysis in 269,867 individuals identifies new genetic and functional links to intelligence. Nat Genet. 2018 Jul;50(7):912–9.

For peer review only

65. Okbay A, Beauchamp JP, Fontana MA, Lee JJ, Pers TH, Rietveld CA, et al. Genome-wide association study identifies 74 loci associated with educational attainment. Nature. 2016 May 26;533(7604):539–42.
66. Cross-Disorder Group of the Psychiatric Genomics Consortium. Electronic address: plee0@mgh.harvard.edu, Cross-Disorder Group of the Psychiatric Genomics Consortium. Genomic Relationships, Novel Loci, and Pleiotropic Mechanisms across Eight Psychiatric Disorders. Cell. 2019 Dec 12;179(7):1469-1482.e11.
67. Ge T, Chen C-Y, Ni Y, Feng Y-CA, Smoller JW. Polygenic prediction via Bayesian regression and continuous shrinkage priors. Nat Commun. 2019 Apr 16;10(1):1776.
68. Centre for Epidemiology. The Swedish Medical Birth Register: summary of content and quality: 2003 [Internet]. [cited 2022 Sep 15]. Available from: https://www.socialstyrelsen.se/globalassets/sharepoint-dokument/artikelkatalog/ovrigt/2003-112-3_20031123.pdf
69. Ludvigsson JF, Almqvist C, Bonamy A-KE, Ljung R, Michaëlsson K, Neovius M, et al. Registers of the Swedish total population and their use in medical research. Eur J Epidemiol. 2016 Feb;31(2):125–36.
70. Ludvigsson JF, Svedberg P, Olén O, Bruze G, Neovius M. The longitudinal integrated database for health insurance and labour market studies (LISA) and its use in medical research. Eur J Epidemiol. 2019 Apr;34(4):423–37.
71. Statistiska centralbyrån SCB. Befolkningens utbildning (Utbildningsregistret, UREG) [Internet]. 2011. Available from: https://www.scb.se/contentassets/776fd7a4ac2f4574ae86e4a3b5b7595e/uf0506_do_2011.pdf
72. Ludvigsson JF, Andersson E, Ekbom A, Feychting M, Kim J-L, Reuterwall C, et al. External review and validation of the Swedish national inpatient register. BMC Public Health. 2011 Jun 9;11:450.
73. Wettermark B, Hammar N, Fored CM, Leimanis A, Otterblad Olausson P, Bergman U, et al. The new Swedish Prescribed Drug Register--opportunities for pharmacoepidemiological research and experience from the first six months. Pharmacoepidemiol Drug Saf. 2007 Jul;16(7):726–35.
74. Wallerstedt SM, Wettermark B, Hoffmann M. The First Decade with the Swedish Prescribed Drug Register - A Systematic Review of the Output in the Scientific Literature. Basic Clin Pharmacol Toxicol. 2016 Nov;119(5):464–9.
75. Brooke HL, Talbäck M, Hörnblad J, Johansson LA, Ludvigsson JF, Druid H, et al. The Swedish cause of death register. Eur J Epidemiol. 2017 Sep;32(9):765–73.
76. Wallert J, Boberg J, Kaldo V, Mataix-Cols D, Flygare O. Predicting remission after internet-delivered psychotherapy in patients with depression using machine learning and multi-modal data. medRxiv [Internet]. 2021; Available from: https://www.medrxiv.org/content/10.1101/2021.04.30.21256367v1.abstract
77. Wallert, J, Boberg, J, Kravchenko, O, Kaldo, V, Mataix-Cols, D, Crowley, J, Rück, C. Polygenic Risk Scores, Data-Driven Longitudinal Clusters of Weekly Symptom Trajectories, and Post Psychotherapy Remission in Major Depressive Disorder. World Congress of Psychiatric Genetics; 2022 Sep 15; Florence, Italy.
78. Gogoulou E, Boman M, Ben Abdesslem F, Hentati Isacsson N, Kaldo V, Sahlgren M. Predicting Treatment Outcome from Patient Texts:The Case of Internet-Based Cognitive Behavioural Therapy. In: Proceedings of the 16th Conference of the European Chapter of the Association for Computational Linguistics: Main Volume. Online: Association for Computational Linguistics; 2021. p. 575–80.

For peer review only

79. Boman M, Ben Abdesslem F, Forsell E, Gillblad D, Görnerup O, Isacsson N, et al. Learning machines in Internet-delivered psychological treatment. Progress in Artificial Intelligence. 2019 Dec 1;8(4):475–85.
80. Schibbye P, Ghaderi A, Ljótsson B, Hedman E, Lindefors N, Rück C, et al. Using early change to predict outcome in cognitive behaviour therapy: exploring timeframe, calculation method, and differences of disorder-specific versus general measures. PLoS One. 2014 Jun 24;9(6):e100614.
81. Clements CC, Karlsson R, Lu Y, Juréus A, Rück C, Andersson E, et al. Genome-wide association study of patients with a severe major depressive episode treated with electroconvulsive therapy. Mol Psychiatry [Internet]. 2021 Jan 22; Available from: https://psycnet.apa.org/fulltext/2021-11534-001.pdf
82. Forstner AJ, Awasthi S, Wolf C, Maron E, Erhardt A, Czamara D, et al. Genome-wide association study of panic disorder reveals genetic overlap with neuroticism and depression. Mol Psychiatry [Internet]. 2019 Nov 11; Available from: http://dx.doi.org/10.1038/s41380-019-0590-2
83. Davies MR, Kalsi G, Armour C, Jones IR, McIntosh AM, Smith DJ, et al. The Genetic Links to Anxiety and Depression (GLAD) Study: Online recruitment into the largest recontactable study of depression and anxiety. Behav Res Ther. 2019 Dec;123:103503.
84. Penninx BWJH, Eikelenboom M, Giltay EJ, van Hemert AM, Riese H, Schoevers RA, et al. Cohort profile of the longitudinal Netherlands Study of Depression and Anxiety (NESDA) on etiology, course and consequences of depressive and anxiety disorders. J Affect Disord. 2021 May 15;287:69–77.
85. Maret-Ouda J, Tao W, Wahlin K, Lagergren J. Nordic registry-based cohort studies: Possibilities and pitfalls when combining Nordic registry data. Scand J Public Health. 2017 Jul;45(17_suppl):14–9.
86. Wallert J, Gustafson E, Held C, Madison G, Norlund F, von Essen L, et al. Predicting Adherence to Internet-Delivered Psychotherapy for Symptoms of Depression and Anxiety After Myocardial Infarction: Machine Learning Insights From the U-CARE Heart Randomized Controlled Trial. J Med Internet Res. 2018 Oct 10;20(10):e10754.

For peer review only

Figure legends

Figure 1. MULTI-PSYCH data structure and research aims. ICBT=Internet-based cognitive behaviour therapy, SNP=Single nucleotide polymorphism, GWAS=Genome wide association study

Figure 2. Data sources. LISA=Longitudinal integrated database for health insurance and labour market studies, VAL=Stockholm County health care register, IPSY=Internet psychiatry clinic.

Figure 3. Available data. The number of individuals providing data from different data sources.

For peer review only

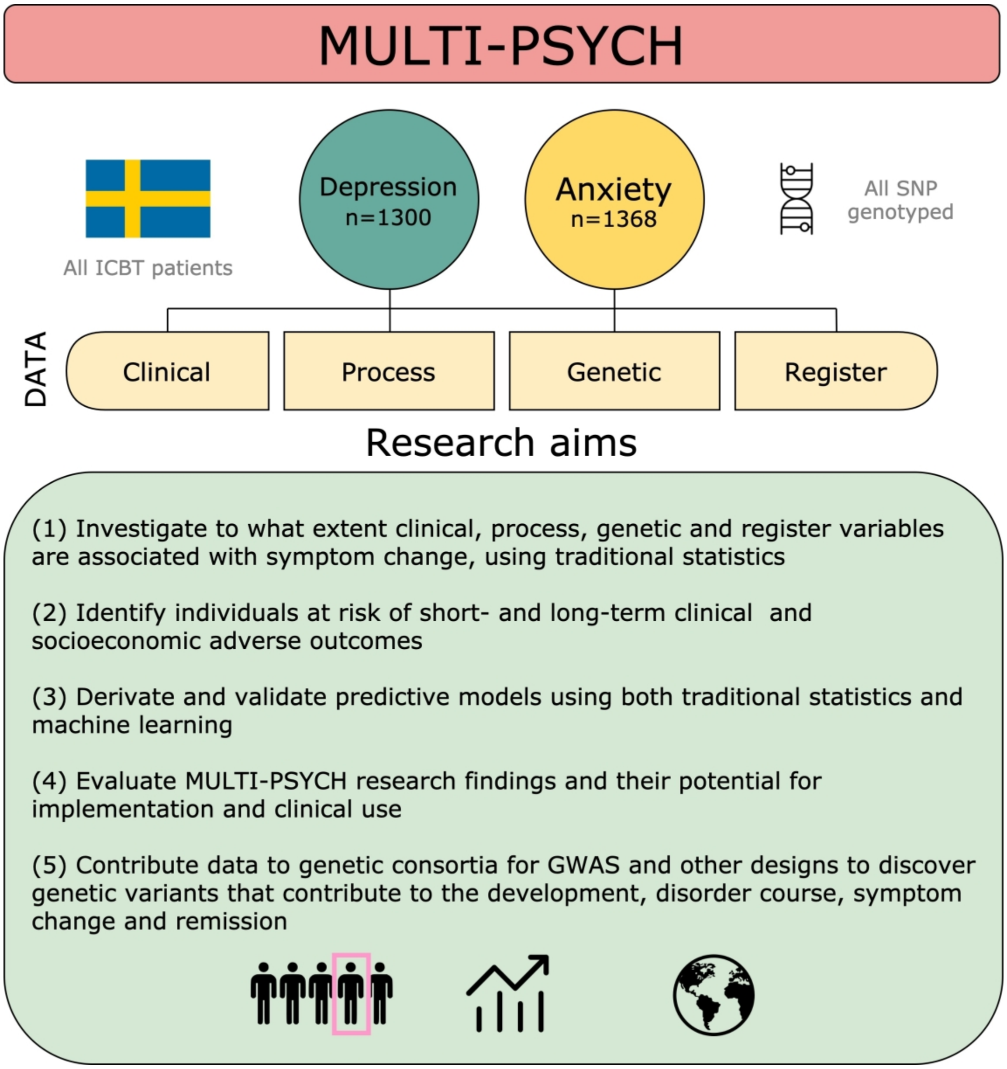

MULTI-PSYCH data structure and research aims. ICBT=Internet-based cognitive behaviour therapy, SNP=Single nucleotide polymorphism, GWAS=Genome wide association study

163x172mm (400 x 400 DPI)

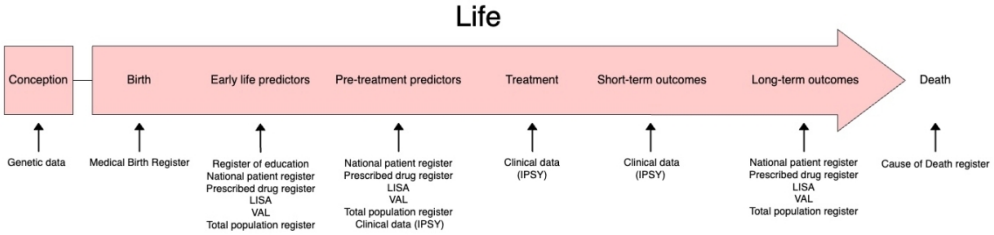

Data sources. LISA=Longitudinal integrated database for health insurance and labour market studies, VAL=Stockholm County health care register, IPSY=Internet psychiatry clinic.

483x121mm (72 x 72 DPI)

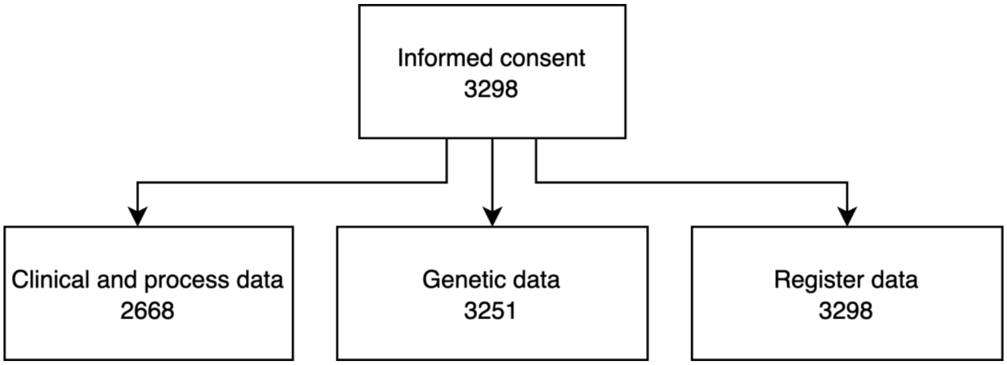

Available data. The number of individuals providing data from different data sources.

114x40mm (400 x 400 DPI)
